# Fluorine‐18‐Labeled Nucleotide Analogs Targeting Ecto‐5'‐Nucleotidase (CD73) for Positron Emission Tomography Imaging of Solid Tumors

**DOI:** 10.1002/anie.202522758

**Published:** 2026-03-15

**Authors:** Clemens Dobelmann, Constanze C. Schmies, Georg Wilhelm Rolshoven, Mirko Scortichini, Stefan Wagner, Andreas Isaak, Riham M. Idris, Jennifer Dabel, Lucie Grey, Karolina Losenkova, Susanne Moschütz, Haneen Al Hroub, Antje Keim, Sandra Höppner, Jouko Sandholm, Pia Boström, Maija Hollmén, Norbert Sträter, Sven Hermann, Gennady G. Yegutkin, Kenneth A. Jacobson, Sonja Schelhaas, Christa E. Müller, Anna Junker

**Affiliations:** ^1^ European Institute for Molecular Imaging (EIMI) University of Muenster Münster Germany; ^2^ PharmaCenter Bonn Pharmaceutical Institute Pharmaceutical & Medicinal Chemistry University of Bonn Bonn Germany; ^3^ Molecular Recognition Section, Laboratory of Bioorganic Chemistry National Institute of Diabetes and Digestive and Kidney Diseases National Institutes of Health Bethesda Maryland USA; ^4^ Department of Nuclear Medicine University Hospital Muenster Münster Germany; ^5^ Institut Für Pharmazeutische und Medizinische Chemie University of Muenster Muenster Germany; ^6^ Medicity Research Laboratory and InFLAMES Flagship University of Turku Turku Finland; ^7^ Institute of Bioanalytical Chemistry Center for Biotechnology and Biomedicine Leipzig University Leipzig Germany; ^8^ Turku Bioscience Centre University of Turku and Åbo Akademi University Turku Finland; ^9^ Department of Pathology University of Turku and Turku University Central Hospital Turku Finland; ^10^ Werner Siemens Imaging Center, Eberhard Karls University of Tuebingen, Department of Preclinical Imaging and Radiopharmacy; Cluster of Excellence iFIT (EXC 2180) Image‐Guided and Functionally Instructed Tumor Therapies Tuebingen Germany

## Abstract

Ecto‐5’‐nucleotidase (CD73) is a potential new drug target for cancer immunotherapy. Its overexpression is associated with various aggressive cancers, including triple‐negative breast cancer (TNBC) and pancreatic cancer, making it a promising target for diagnostic imaging. Besides antibodies, small‐molecule CD73 inhibitors have been developed and are currently in clinical trials. This study aimed to develop and evaluate fluorine‐18 labeled high‐affinity CD73 inhibitors as tracers for the non‐invasive positron emission tomography (PET) imaging of CD73 expression in cancer. Two CD73 inhibitors were selected for radiolabeling based on their high potency (K_i_ values of ca. 1 nM) and favorable pharmacokinetic properties, yielding [^18^F]PSB‐19427 ([^18^F]**1**) and [^18^F]MRS‐4648 ([^18^F]**2**). Ex vivo imaging studies on human breast cancer tissues indicated specific binding of both radiotracers. Subsequent in vivo studies proved [^18^F]**1** to be superior due to its long elimination half‐life and its accumulation in TNBC and pancreatic cancer tissues, suggesting its potential as a versatile PET tracer for imaging of various solid tumors. Compared to [^18^F]FDG, [^18^F]**1** was superior in visualizing TNBC, offering potential advantages over [^18^F]FDG in terms of specificity and diagnostic accuracy. Thus, [^18^F]**1** is a PET tracer with outstanding properties suitable for broad application in cancer diagnosis and personalized medicine.

## Graphical abstract

Fluorine‐18‐labeled CD73 inhibitor [^1^ ^8^F]PSB‐19427 enables noninvasive PET mapping of CD73 overexpression in triple‐negative breast (TNBC) and pancreatic cancer. For [^1^ ^8^F]PSB‐19427, specific binding in human tumor tissues, favorable biodistribution with prolonged blood retention, robust tumor uptake, and superiority to [^1^ ^8^F]FDG in a TNBC model, supporting clinical translation for patient stratification and immuno‐oncology, were demonstrated.

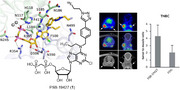

## 1 Introduction

Human ecto‐5’‐nucleotidase, also termed cluster of differentiation 73 (CD73), is a ubiquitously expressed homodimeric enzyme that, exists in soluble and glycosylphosphatidylinositol (GPI)‐anchored membrane‐bound form. In addition, it is found on the surface of exosomes [1]. CD73 catalyzes the extracellular hydrolysis of nucleoside monophosphates, yielding the corresponding nucleosides. CD73 converts extracellular adenosine monophosphate (AMP) to immunosuppressive adenosine and inorganic phosphate [2, 3]. AMP is generated from pro‐inflammatory ATP or other adenine nucleotides [4, 5]. Due to the hypoxic tumor microenvironment (TME), the NT5E gene encoding for CD73 is upregulated in cancer cells in a hypoxia‐inducible factor 1α (HIF1α)‐dependent manner [6]. Additionally, upregulated pro‐inflammatory factors, such as transforming growth factor β (TGF‐β), interferons (IFNs), tumor necrosis factor (TNF), interleukin‐1β (IL‐1β), and the wingless‐related integration site (Wnt)/β‐catenin pathway further promote CD73 expression [7]. The enzyme is expressed on cytotoxic CD8^+^ T‐cells [1] and regulatory T cells (Tregs) [8] and is overexpressed in various cancer types, including melanoma, bladder, kidney, colon, ovarian, pancreatic, and breast cancer [9, 10]. It was shown to promote cancer cell migration, invasion, epithelial‐mesenchymal transition (EMT), and possibly contribute to chemotherapy resistance [11, 12, 13, 14]. Several studies demonstrated the prognostic value of CD73 expression in triple‐negative breast cancer (TNBC) [12, 15], pancreatic cancer [16], and lung adenocarcinoma [17]. Various clinical trials (phases I to III) with small‐molecule CD73 inhibitors (AB680/Quemliclustat, LY3475070) and monoclonal anti‐CD73 antibodies (MEDI9447/Oleclumab, NZV930/SRF373, TJ004309/TJD5, CPI‐006, and BMS‐986179) are ongoing [18, 19, 20]. Additionally, [^68^Ga]Ga‐labeled DOTA‐dPNE is currently in phase I clinical trials for breast cancer imaging (NCT06844110), and a [^8^ ^9^Zr]‐labeled anti‐CD73 IgG has demonstrated high specificity in xenograft mouse models of colon cancer [21]. In recent years, based on our lead structures PSB‐12379 [22] and PSB‐12489 [23], a variety of nucleotide‐derived CD73 inhibitors with nanomolar potency at rodent and human CD73, high selectivity, and high metabolic stability have been developed and tested as potential anticancer drugs [24, 25, 26, 27, 28]. Given the general role of CD73 in cancer development and progression and encouraging results of antibody‐derived CD73‐targeting tracers [21], we have been interested in the diagnostic potential of positron emission tomography (PET) imaging of CD73 expression in TNBC and pancreatic cancer using small‐molecule‐based tracers.

Breast cancers show a very high incidence, comprising ca. 12% of all cancers [29]. TNBC accounts for 10%–20% of all breast cancers and exhibits an especially aggressive clinical progression [30]. Early and precise detection of breast cancer and its metastases is expected to enhance the survival rate. Especially for TNBC, many standard imaging methods, such as mammography and ultrasound, fail since detectable abnormalities are often missed, whereas CD73 is considered a promising biomarker for TNBC [12, 31].

Due to its asymptomatic progression at early stages and its tendency to form distant metastases early on, pancreatic cancer is particularly aggressive and among the deadliest cancers (5‐year survival rate <9%) [32]. Despite significant effort, there is still no effective drug available for pancreatic cancer therapy, and the only potentially curative treatment to date is its complete surgical resection. Hence, the key to optimal therapy management is tumor detection at a very early stage. PET with 2‐deoxy‐2‐[^18^F]fluoro‐d‐glucose ([^18^F]FDG), the commonly employed radiotracer for imaging pancreatic cancer, leads to 90% overall diagnostic accuracy but has low spatial resolution. Moreover, false‐positive signals caused by physiologic FDG uptake limit the detection of small metastases [33]. Thus, alternative and more reliable tumor markers are urgently needed for the successful diagnosis of pancreatic cancer. Most recently, encouraging preclinical and clinical results of phase I to III studies of CD73 inhibitor AB680 (Quemliclustat) in pancreatic cancer were reported (NCT03677973, NCT04575311 NCT04104672, NCT05915442, and NCT06608927). A phase III clinical study of Quemliclustat and chemotherapy versus placebo and chemotherapy in patients with metastatic pancreatic ductal adenocarcinoma is currently ongoing (NCT06608927), highlighting the great potential of targeting CD73 in pancreatic cancer.

Thus, CD73 appears to be a promising biomarker for both TNBC and pancreatic cancer, warranting in‐depth evaluation via PET imaging for primary cancer diagnosis and patient selection for CD73‐targeted immunotherapy. In the present study, we developed two structurally diverse fluorinated CD73 inhibitors, the adenine‐based PSB‐19427 (**1**) and the cytosine‐based MRS‐4648 (**2**), and evaluated them as PET tracers for imaging CD73 expression in solid tumors. To perform a broad characterization, complementary biochemical, structural, tissue‐based, and in vivo imaging approaches were pursued.

## 2 RESULTS and DISCUSSION

### 2.1 Design and Development of PET Tracers

**SCHEME 1:**
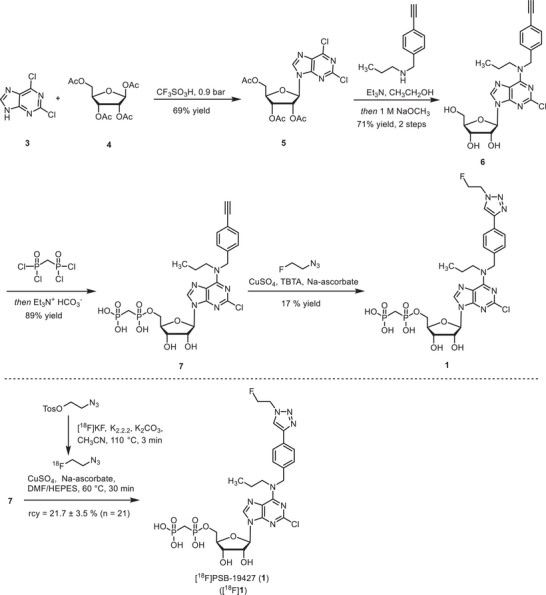
Synthesis of precursor **7**, CD73 inhibitor PSB‐19427 (**1**), and radiosynthesis of [^18^F]**1**. TBTA: tris((1‐benzyl‐4‐triazolyl)methyl)amine, DMF: dimethylformamide, and HEPES: 4‐(2‐hydroxyethyl)‐1‐piperazineethanesulfonic acid.

**SCHEME 2:**
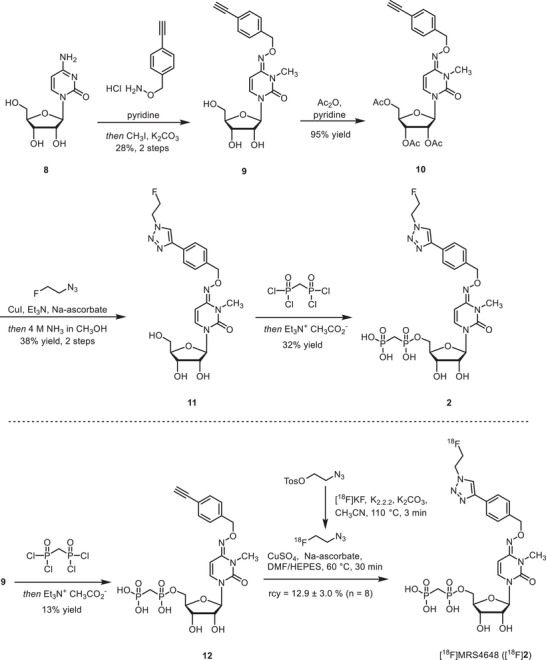
Synthesis of precursor **12**, CD73 inhibitor MRS‐4648 (**2**), and radiosynthesis of [^18^F]**2**.

**FIGURE 1:**
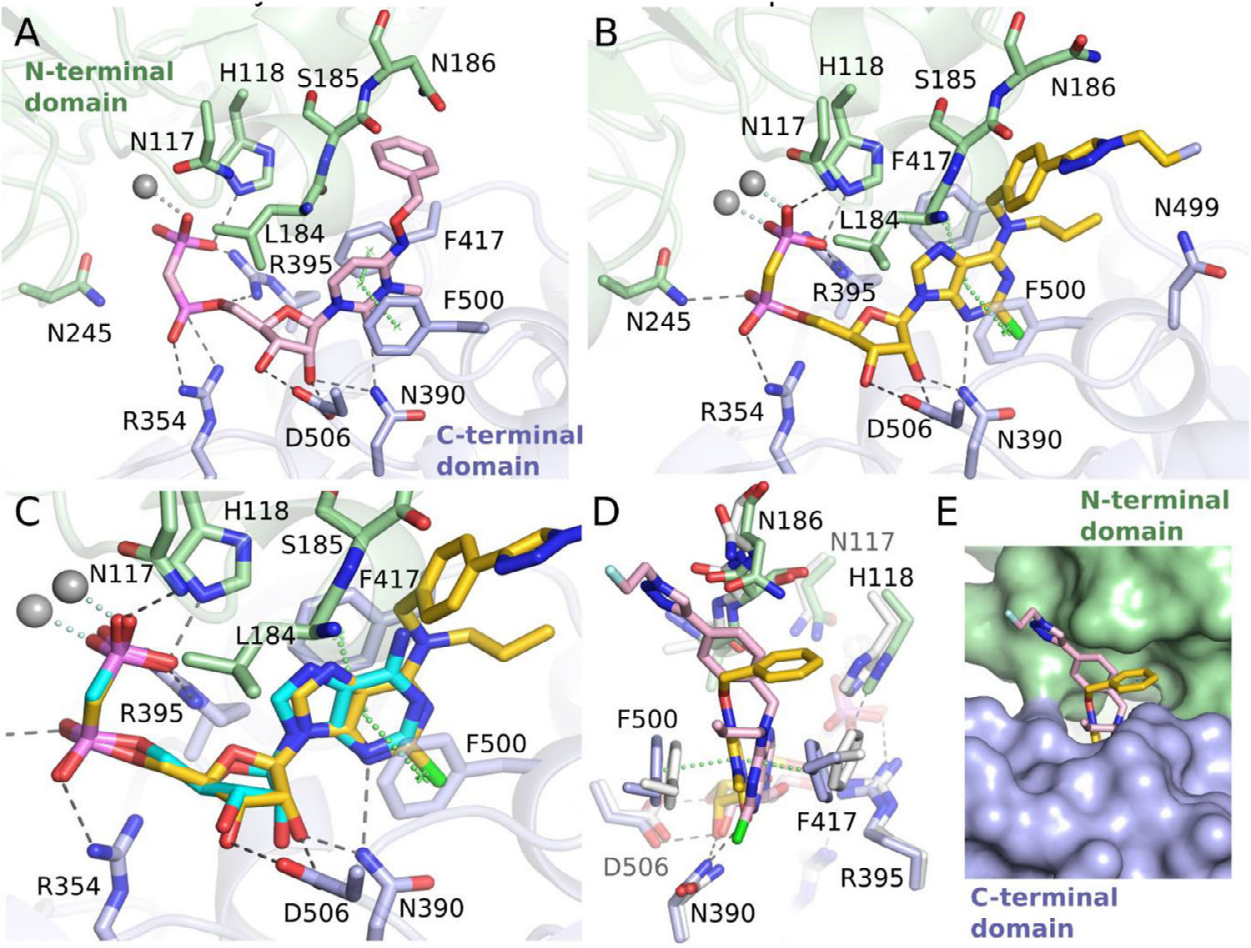
X‐ray crystal structure analysis. (A). Binding mode of JMS04‐14 (**14**) to CD73. (B). Binding mode of PSB‐19427 (**1**) in the active site of CD73. Only selected residues interacting with **1** via polar or hydrophobic contacts are shown. Gray spheres indicate the two zinc ions. (C). Superposition of CD73×**1** (yellow) and CD73×AMPCP (**13**, cyan, PDB: 4H2I). (D). Superposition of CD73×PSB‐19427 (yellow) with CD73×**14** (light pink) based on residues of both domains.(E). Solvent accessibility of the substituents for attachment of the radiolabels. The molecular surface of CD73 is shown with the binding modes of **1** (yellow) and **14** (light pink)[35].

Building on our previous structure‐activity relationship studies [9, 22, 23, 26, 27, 34] we designed two structurally diverse inhibitors, PSB‐19427 (**1**, K*_i _* = 1.02 ± 0.11 nM, human CD73, Scheme 1) and MRS‐4648 (**2**, K*_i_* = 0.664 ± 0.089 nM, human CD73, Scheme 2). Our design prioritized high CD73 affinity and the ability to incorporate a fluorine‐18 atom in the final reaction step. The design of **1** was guided by the high tolerance of the CD73 enzyme for substituents at the *p*‐position of the *N^6^*‐benzylamino group. This had previously been demonstrated by co‐crystal structures of CD73 with AMPCP derivatives with a benzyl substituent at the *N^6^*‐position of the adenine core, such as PSB‐12489 (pdb id 6S7H) [23]. The design of compound **2** was guided by the binding mode of compound JMS04‐14 (**14**), for which we obtained a co‐crystal structure at 2.55 Å resolution (Figure 1A, Table S1). These studies indicated similar tolerability of bulky substituents at the *p*‐position of the phenyl ring attached to the nucleobase (Figure 1D). Since both inhibitors carried a diphosphonate group, we selected a Huisgen cycloaddition reaction using [^18^F]fluoroethyl azide as a labeling strategy. The advantage of this procedure is that, the acidic phosphonate group in precursors **7** and **12** does not come into contact with the nucleophilic fluoride during the radiolabeling reaction. In a direct fluorination reaction via nucleophilic substitution, this interaction would have led to protonation of the fluoride, thereby hindering the radiolabeling process.

The synthesis of compound **1** started from the formation of the protected nucleoside **5**, which was subsequently reacted with *N*‐(4‐ethynylbenzyl)propan‐1‐amine to afford nucleoside **6** in 71% yield. Reaction of **6** with methylenebis(phosphonic dichloride) afforded the phosphorylated product **7** in 89% yield. Huisgen cycloaddition with fluoroethyl azide led to the formation of compound **1**.

For the synthesis of compound **2**, cytidine (**8**) was reacted with *O*‐(4‐ethynylbenzyl)hydroxylamine‐HCl in pyridine, followed by the introduction of the methyl group at the three‐position of the pyrimidine ring. Protection of the hydroxyl groups, followed by Huisgen cycloaddition with fluoroethyl azide and subsequent cleavage of the protective groups, led to the formation of nucleoside **11** in 38% yield. Subsequent phosphorylation using methylenebis(phosphonic dichloride) afforded compound **2** in 32% yield.

Compounds **1** and **2** were characterized as potent competitive inhibitors of human CD73 with K*_i_* values of around 1 nM, determined in an enzyme inhibition assay using soluble recombinant human CD73 [36]. For **1**, CD73 affinity was additionally determined at rat CD73 (K*_i_* = 6.09 ± 0.74 nM, rat soluble CD73) and mouse CD73 (K*_i_* = 47.8 ± 10.5 nM, mouse membrane‐bound CD73). Both compounds were evaluated for metabolic stability in mouse liver microsomes (MLM) and plasma protein binding (PPB) (Table 1). PSB‐19427 (**1**) displayed higher PPB (>99%) and slightly lower microsomal stability (17 ± 2% degradation in MLM after 90 min incubation) in comparison to MRS‐4648 (**2**, 66% PPB, 3% ± 1% degradation).

**TABLE 1:** In vitro assay data, metabolic stability in mouse liver microsomes (MLM), plasma protein binding (PPB), and log*D*_7.4_‐values of **1** and **2**.

Target/Assay/Property	1	2
Human soluble CD73^[^ ^a^ ^]^	K*_i_* = 1.02 ± 0.11 nM	K*_i_* = 0.664 ± 0.089 nM
Human membrane‐bound CD73^[^ ^b^ ^]^	K*_i _* = 2.78 ± 0.47 nM	n.d.^[^ ^f^ ^]^
Rat soluble CD73^[^ ^c^ ^]^	K*_i _* = 6.09 ± 0.74 nM	n.d.
Mouse membrane‐bound CD73^[^ ^d^ ^]^	K*_i_* = 47.8 ± 10.5 nM	n.d.
Plasma protein binding (PPB)	>99%	66%
Metabolic stability in mouse liver microsomes (MLM)^[^ ^e^ ^]^	17 ± 2%	3 ± 1%
log*D*_7.4_	−0.12 ± 0.03	0.74 ± 0.3

^a^ Soluble recombinant human CD73 (K_m_ 17 µM, substrate AMP (5 µM)).

^b^ Membrane preparation of human TNBC (MDA‐MB‐231 cell line, K_m_ 14.8 µM, substrate AMP (5 µM)).

^c^ Soluble recombinant rat CD73 (K_m_ 59 µM, substrate AMP (5 µM)).

^d^ Membrane preparation of mouse 4T1.2 breast cancer cell line (K_m_ 67.6 µM, substrate AMP (5 µM)).

^e^ MLM: mouse liver microsomes. MLM stability: % decomposition in MLM preparation after 90 min of coincubation.

^f^n .d. = not determined.

Due to its structural similarity to ADP, the lead compound **1** was additionally profiled for selectivity versus the ADP‐activated P2Y receptors P2Y_1_ and P2Y_12_; these data confirmed its high selectivity for CD73 and are provided in the Supporting Information (Tables S4 and S5).

### 2.2 X‐Ray Co‐Crystal Structure

To further characterize CD73 interactions with **1**, we determined a co‐crystal structure (Figure 1B, Table S1). The inhibitor **1** adopts a binding mode in the closed conformation of CD73 similar to that of other AMPCP derivatives, the large *N* ^6^‐substituent interacting with the N‐terminal domain. In comparison to the CD73×AMPCP complex in the closed state in crystal form III [37] the adenine ring of PSB‐19427 is rotated slightly such that the N^6^ atom shifts by about 1.0 Å toward the C‐terminal domain (Figure 1C). We also superimposed PSB‐12489 [23], which structurally resembles compound **1** (Figure S6A). The benzyl groups at *N* ^6^ of the two derivatives show a close agreement in their binding modes. It is important to note that, the electron density around the *N* ^6^‐substituent of **1** is significantly weaker than for the rest of the structure (Figure S6B). This finding indicates high flexibility of this substituent, which has previously been observed for other co‐crystal structures of CD73 with *N* ^6^‐substituted adenine nucleotide analogs [23, 24]. Interestingly, the triazole group is better defined in the electron density than the phenyl ring in both chains of the asymmetric unit, and it forms nonpolar interactions with Asn186. This interaction may contribute to the compound's binding properties. Superimposing inhibitor **1** and JMS04‐14 (**14**) reveals that, the phenyl rings to which the radiolabels are attached are oriented differently within the cleft between the two protein domains. However, both positions offer sufficient space for the attachment of bulky substituents (see Figure 1D, E). The co‐crystal structure provides direct molecular evidence that compound **1** binds to the catalytic site of CD73 in a defined and specific manner.

### 2.3 Radiosynthesis

CD73 inhibitors **1** and **2** were subsequently prepared in ^18^F‐labeled form. The alkyne‐substituted precursors **7** and **12** were subjected to an azide‐alkyne Huisgen‐cycloaddition reaction using ^18^F‐labeled fluoroethyl azide (Schemes 1 and 2). [^18^F]**1** was obtained in 21.7% ± 3.5% radiochemical yield (rcy), with >99% radiochemical purity (rcp) and a molar activity (*A*_m_) of 2.3‐54.3 GBq/µmol (*n* = 21). The compound [^18^F]**2** was isolated in 12.9 ± 3.0 % rcy, with >98% rcp and a molar activity of 0.4‐6.3 GBq/µmol (*n* = 8). Radiochemical identity was confirmed by observing comobility upon spiking with authentic samples of **1** and **2** (Figure S1 and S2), respectively, in radio‐high‐performance chromatography. Both tracers displayed high affinity to glass surfaces, but were sufficiently soluble in water for injection (WFI)/ethanol (9:1) at concentrations required for imaging applications (50–150 MBq/mL).

### 2.4 Lipophilicity (log*D*_7.4_) and Plasma Stability of Radiotracers

The log*D*_7.4_ values of both radiotracers were determined using a previously described method based on their distribution between phosphate‐buffered saline (PBS) and octan‐1‐ol [38, 39]. This revealed a higher polarity of the purine‐based [^18^F]**1**; log*D*_7.4 _= ‐0.12 ± 0.03 as compared to the pyrimidine‐derived [^18^F]**2,** log*D*_7.4 _= 0.74 ± 0.29. Both tracers were found to be stable for at least 90 min at room temperature in human and mouse plasma (100%, see Figure S3).

### 2.5 CD73 inhibitors 1 and 2 Bind to CD73 in Breast Cancer Tissues with High Affinity and Inhibit Enzymatic Activity

**FIGURE 2:**
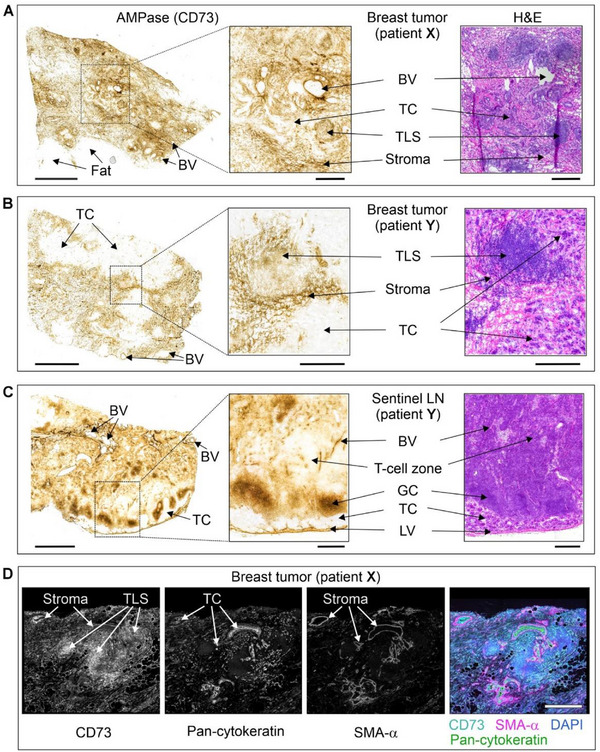
Tissue‐specific distribution of CD73 in breast tumor and lymph nodes. The primary tumors and sentinel lymph nodes (LN) were obtained from two patients with hormone‐receptor‐positive/Her2‐negative grade II infiltrating ductal carcinoma (patient **X**), and hormone‐receptor‐negative/Her2‐positive grade III infiltrating ductal carcinoma with micropapillary differentiation (patient **Y**). CD73 activity was assayed by incubating the cryosections of breast tumors from patients **X** (A) and **Y** (B), and also sentinel LN from patient **Y** (C) with 400 µM AMP in the presence of Pb(NO_3_)_2_, followed by microscopic detection of AMP‐derived inorganic phosphate (P_i_) as a brown precipitate of formed Pb_3_(PO_4_)_2_. Tissue sections were also stained with hematoxylin and eosin (H&E). (D) For immunofluorescence staining, the tumor section from patient **X** was co‐stained with an anti‐CD73 antibody, together with a marker of epithelial cancer cells (pan‐cytokeratin) and the stromal marker α‐smooth muscle actin (SMA‐α). Single channels are shown in grayscale, and the right panel displays a merged image with nuclei counterstained with DAPI. Abbreviations: BV, blood vessels; GC, germinal center; LV, lymphatic vessels; TC, tumor cells, and TLS, tertiary lymphoid structure. Scale bars, 2 mm (A–C) and 500 µm (A–C, right insets, D).

The binding of the non‐radioactive CD73 inhibitors was further evaluated in primary breast tumor tissues and sentinel lymph nodes (LN) surgically removed from two patients with hormone receptor‐positive/Her2‐negative grade II infiltrating ductal carcinoma (patient **X**) and hormone receptor‐negative/Her2‐positive grade III infiltrating ductal carcinoma with micropapillary differentiation (patient **Y**). Tissue‐specific distribution of AMPase activity (hydrolysis of AMP to adenosine and phosphate) and expression levels of CD73 in the breast tumors and the TME were determined by lead nitrate‐based enzyme histochemistry, where the lead phosphate that precipitated due to CD73 activity was visualized as a brown deposit (Figure 2A–C), and by immunofluorescence staining (Figure 2D), respectively [40]. Additional staining of the tissue cryosections with hematoxylin and eosin enabled the visualization of the main histological structures (Figures 2A–C, right insets). Both AMPase activity and CD73 immunoreactivity were primarily associated with SMA‐α^+^ stromal cells, tertiary lymphoid structures (TLS, mainly comprised of T‐ and B‐cell aggregates [41]), blood and lymphatic vessels, and B‐cell zone (in case of sentinel LN dissected from patient **Y**), but not with pan‐cytokeratin‐positive tumor cells themselves.

**FIGURE 3:**
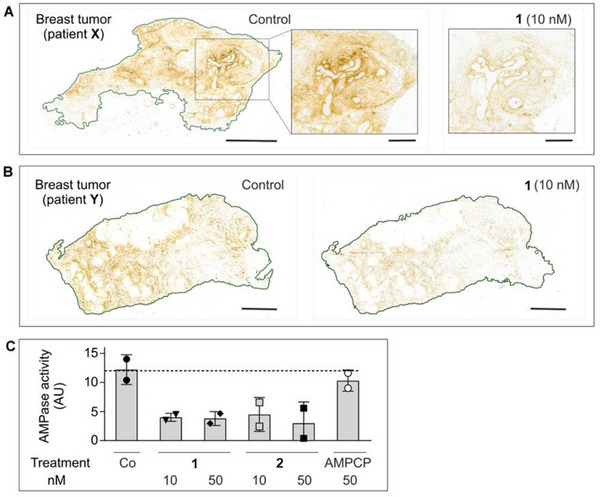
Effects of CD73 inhibitors on AMPase activity in human breast tumor tissues. The effects of CD73 inhibitors on AMPase activity was determined in situ by incubating primary breast tumor samples from patient **X** (A) and patient **Y** (B) with 400 µM AMP and 1.5 mM Pb(NO_3_)_2_ in the absence (control) and the presence of the indicated concentrations of CD73 inhibitor. (C). Mean pixel intensities of brown staining due to the hydrolysis of AMP leading to Pb_3_(PO_4_)_2_ precipitation were quantified in the selected regions and expressed as arbitrary units (AU) (mean ± SEM; *n* = 2). Scale bars: 2 mm (A, B), and 500 µm (A, right insets).

Subsequent analysis of CD73‐mediated AMPase activities was performed by incubating tissue cryosections with AMP and CD73 inhibitors at different concentrations. Figure 2 depicts representative images of AMP‐specific staining in breast tumors from patient **X** (panel A) and patient **Y** (panel B), determined in the absence (control) and in the presence of 10 nM of the potent CD73 inhibitor **1**. Treatment of tissue cryosections with increasing concentrations of **1** and **2** (10–50 nM), but not with the employed concentration of the classical, much less potent CD73 inhibitor AMPCP (**13**, 50 nM), reduced CD73/AMPase activity in the breast TME by ∼60%–70% (Figure 3C).

Based on this encouraging data, we decided to proceed with both compounds to autoradiography and in vivo PET imaging studies.

### 2.6 Autoradiography of [^18^F]1 and [^18^F]2 in Human Breast Cancer Samples

**FIGURE 4:**
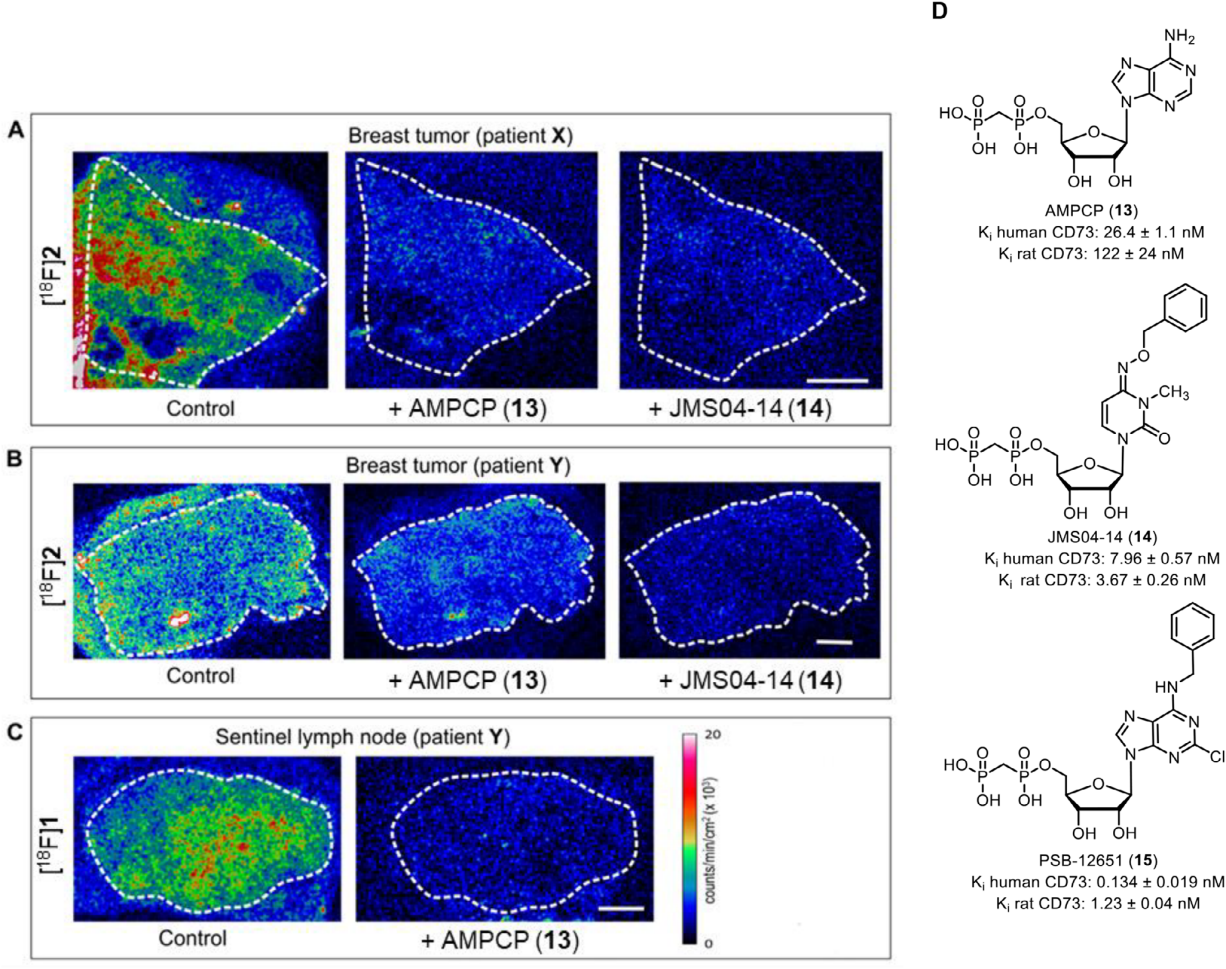
Autoradiographic imaging of ^18^F‐labeled CD73 tracer binding to tissue samples of breast tumors and sentinel lymph nodes. Primary breast tumor (A, B) and LN cryosections (C) were incubated with [^18^F]**2** or [^18^F]**1** in the absence (control) and in the presence of an unlabeled CD73 inhibitor, AMPCP (**13**) or JMS04‐14 (**14**), as indicated. The samples were processed for autoradiographic analysis, as described in the materials and methods section. The white dotted lines outline the tissue boundaries. (D). Structures of CD73 inhibitors applied as blockers in this study.

To test specific radiotracer binding to its target, we performed in situ autoradiography. [^18^F]**2** was applied onto cryosections of neighboring slides of tumor tissues from primary breast tumors from patient **X** (Figure 4A) and patient **Y** (Figure 4B), used for immunohistochemistry and AMPase activity determination. Sentinel LN from patient **Y** was incubated with [^18^F]**1** (Figure 4C). In all three cases, a significant accumulation of radiotracer was detected in the breast tumor tissue (Figure 4A–C, left panels). Notably, residual activity was also detected outside the tissue boundaries, which presumably reflects the ability of the radiotracers (especially [^18^F]**2**) to bind to the microscope slide. Importantly, co‐incubation of the samples with a ∼1000‐fold excess of the CD73 inhibitor AMPCP (**13**), or our recently developed pyrimidine‐based CD73 inhibitor JMS04‐14 (**14,** Figure 4D) [26] with the radiotracer markedly reduced the amount of tissue‐associated radioactivity (Figure 4A–C). Collectively, these autoradiographic data, when analyzed together with the histological and non‐radioactive AMPase activity assays (Figures 2 and 3), provide evidence for the ability of both tracers, [^18^F]**1** and [^18^F]**2**, to selectively bind to CD73 in human breast cancer samples.

Together, enzymatic histochemistry, immunofluorescence, functional inhibition of AMPase activity, and displaceable autoradiographic binding converge to demonstrate that, compounds **1** and **2** bind selectively to enzymatically active CD73 in human tumor tissue.

### 2.7 Biodistribution Studies of [^18^F]1 and [^18^F]2 in Mice

**FIGURE 5:**
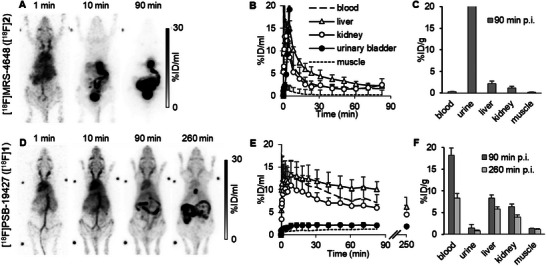
Biodistribution of the investigated CD73 PET tracers. (A–C). Biodistribution of [^18^F]**2** in C57BL/6 WT mice. A. PET maximum intensity projections of a representative mouse (B). Quantification of tracer distribution (*n* = 3) (C). Sum of ex vivo gamma counter experiments (*n* = 3). (D–F). Biodistribution of [^18^F]**1** in C57BL/6 WT mice. (D). Maximum intensity projections of a representative mouse. (E). Quantification of tracer distribution (*n* = 3) (F). Sum of ex vivo gamma counter experiments after 90 min (*n* = 3) and 260 min (*n* = 3).

Next, biodistribution studies of compounds [^18^F]**1** and [^18^F]**2** were performed in female C57BL/6 WT mice. Dynamic (0–90 min), static (77–90 min), and late (240–260 min) scans were performed in a small animal PET scanner. Each PET scan was combined with computer tomography (CT). In the case of [^18^F]**2**, >95% of the tracer was excreted within the first 30 min via renal (19%ID) and hepatobiliary (60%ID, Figure 5A–C) routes. After 90 min, most of the tracer was found in the intestine, gallbladder, bladder (urine), and liver, while only 0.3 ± 0.1 %ID/mL of the radiosignal was detected in the blood at that time point (Figure 5B, C). Concentrations in the lung, heart, muscle, and brain were also relatively low. Nevertheless, during the first 3 min, the tracer was distributed within the whole body, reaching every tissue (Figure 5A).

The biodistribution of [^18^F]**1** was significantly different from that of [^18^F]**2** (Figure 5D–F). The radiotracer [^18^F]**1** was injected into the tail vein, entered the heart, and was subsequently distributed throughout the whole body. The renal excretion route was negligible (2%ID), with the primary excretion route being hepatobiliary (37%ID at 90 min p.i.) The tracer showed long retention in blood, while the concentration in muscle was low (Figure 5E). Nearly no tracer accumulated in brain and urinary bladder, facilitating a favorable signal‐to‐noise ratio. We prolonged the imaging and performed an additional PET scan after 260 min to further increase the signal‐to‐background ratio. In all investigated residual organs (kidney, lung, heart, liver, and spleen), [^18^F]**1** was present even after 260 min, indicating a favorable biodistribution and high metabolic stability in vivo (Figure 5F).

### 2.8 Evaluation of [^18^F]1 and [^18^F]2 in a Mouse MDA‐MB‐231 Breast Cancer Model

**FIGURE 6:**
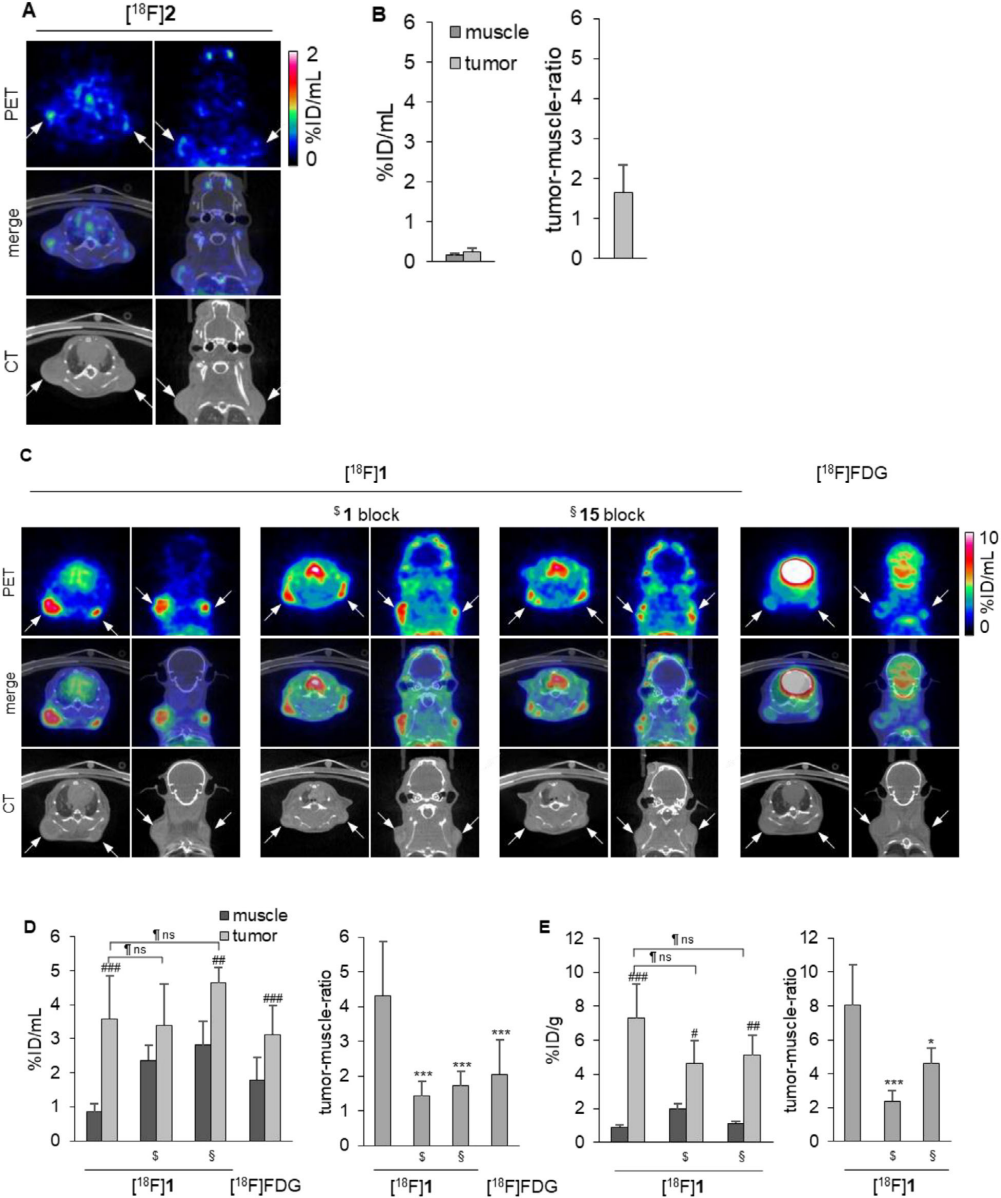
PET imaging data in an MDA‐MB‐231 tumor mouse model. (A). PET images, CT scans, and merged images of [^18^F]**2** in the MDA‐MB‐231 tumor mouse model 90 min after tracer injection. White arrows mark the tumor positions. (B). Quantitative PET analysis of [^18^F]**2** uptake: activity concentration of [^18^F]**2** in muscle and tumor tissues 90 min after tracer injection (left) and respective tumor‐to‐muscle ratio (right, *n* = 5 tumors). (C). PET‐CT images of [^18^F]**1** and [^18^F]FDG in the MDA‐MB‐231 tumor mouse model, 260 min ([^18^F]**1**), or 75 min ([^18^F]FDG) after injection. From left to right: [^18^F]**1** without blocker, pretreating with unlabeled compound **1** (PSB‐19427) or ^§^PSB‐12651 (**15**) 10 min before tracer injection, and [^18^F]FDG. White arrows mark the positions of tumors. (D). Quantitative PET analysis of [^18^F]**1** uptake: activity concentration of [^18^F]**1** and [^18^F]FDG in muscle and tumor tissues (left) and tumor‐to‐muscle ratios (right) after 260 min. [^18^F]**1**: *n* = 18 tumors, [^18^F]**1** plus unlabeled **1** block: *n* = 8 tumors, [^18^F]**1** plus compound **15** block: *n* = 6 tumors, [^18^F]FDG: *n* = 18 tumors E. Ex vivo analysis of [^18^F]**1** uptake as measured by gamma counter: activity concentrations of [^18^F]**1** in muscle and tumor tissues after 260 min without and with blocking (left) and tumor‐to‐muscle ratios (right). [^18^F]**1**: *n* = 6 tumors, [^18^F]**1** plus unlabeled **1** block: *n* = 6 tumors, [^18^F]**1** plus compound **15** block: *n* = 5 tumors.^#^ relative to the respective muscle, *relative to [^18^F]**1** without blocker, relative to the unblocked tumor; *<0.05; **<0.01, ***<0.001,^#^<0.05; ^##^ <0.01, ^###^<0.001, <0.05;  <0.01,  <0.001, ns: non‐significant, unpaired t‐test.

Given the specificity of [^18^F]**1** and [^18^F]**2** and the promising in vivo biodistribution profile of [^18^F]**1**, we established a small‐animal human breast tumor xenograft model in NSG mice subcutaneously implanted with MDA‐MB‐231 cells. This highly invasive triple‐negative breast adenocarcinoma cell line exhibits high CD73 expression and therefore, offers a suitable model for investigating the role of CD73 in TNBC [42, 43]. For blocking studies, PSB‐12651 (**15**, Figure 6) [22] or unlabeled **1** were used.

Similar to the initial radiotracer biodistribution study (Figure 5A), [^18^F]**2** distribution was characterized by rapid liver uptake and clearance via both renal and hepatobiliary routes, with very low tumor accumulation (Figure 5AB). At the end of the study, 90 min *p.i*., most of the tracer was detected in the bladder. Relevant concentrations were also located in the liver, spleen, and kidneys, indicating that the tracer was rapidly excreted.

Unlike [^18^F]**2**, the tracer [^18^F]**1** showed elongated blood retention with sufficient accumulation in the tumor tissues. The tumors were visible in the PET scans after 260 min (Figures. 6C and S4). MDA‐MB‐231 tumor uptake of **1** was at 3.60% ± 1.27%ID/mL, while 0.86% ± 0.24% ID/mL was found in the muscle, reflecting a low background with a corresponding tumor‐to‐muscle ratio of 4.32 ± 1.54 after 260 min. Tumor uptake was relatively stable over more than 4 h (Figures S4 and S5). By pre‐treating the mice with unlabeled **1** or the structurally distinct CD73 inhibitor **15**, the tumor‐to‐muscle ratio was markedly reduced (Figure 6C–E), without a significant reduction in the tumor signal from blocking. Incomplete displacement in blocking experiments is a recognized phenomenon in molecular imaging and does not necessarily indicate nonspecific binding. Notably, similar behavior has been reported for highly specific antibody‐based PET tracers that have been successfully advanced into clinical application. For example, in preclinical and translational studies with the PD‐1–targeting tracer ^8^ ^9^Zr‐pembrolizumab, co‐administration of excess unlabeled antibody markedly reduced tracer uptake in lymphoid organs but did not reduce tumor uptake, despite confirmed target specificity, which did not prevent subsequent clinical use [44, 45]. In a similar manner, incomplete, or absent displacement has also been described for the MAO‐B radioligand ^1^ ^1^C‐l‐deprenyl, which exhibits kinetics dominated by irreversible enzyme binding [46], such that acute blocking produces limited measurable signal reduction despite well‐established target specificity and extensive clinical use.

Besides affinity, binding kinetics play a crucial role in determining in vivo tracer performance. The clinical CD73 inhibitor Quemliclustat has been reported to display a very long residence time [47]. We therefore determined the kinetic rate index (KRI) of selected CD73 inhibitors using a radioligand binding assay. Compounds **1** and **15** were estimated to have residence times of approximately 22 and 31 min, respectively, compared with the reference inhibitor AMPCP (**13**, 8.8 min), determined at 25 °C. These comparatively long residence times indicate slow dissociation from the target, which may contribute to the sustained tumor retention of [^18^F]**1** observed in vivo. Another effect that might play a role is pharmacological inhibition of CD73 by the high concentration of the CD73 inhibitor used for blocking. This will lead to a strong reduction in adenosine concentrations, for example, in blood vessels and other tissues [3], which may change hemodynamics and drug distribution. In our study, the blocking regimen was not further escalated, and the time points between blocker and PET tracer application were not varied; thus, higher inhibitor doses and/or longer pre‐dosing intervals to allow sufficient target engagement may be required to achieve measurable displacement in vivo.

As a next step, we compared the performance of [^18^F]**1** in the MDA‐MB‐231 xenograft mouse model to [^18^F]FDG PET/CT as a clinical diagnostic approach, which is recommended for systemic staging (stages IIB‐IV) of no special type breast cancer [48], despite its limited diagnostic accuracy, in particular in lower stages of disease, due to low tracer uptake and false positive signals [48]. While the MDA‐MB‐231 tumors are only barely visible by [^18^F]FDG imaging (tumor‐to‐muscle ratio of 2.04 ± 1.0, Figure 6C, D), the tumors can be clearly identified in the same animals by [^18^F]**1** imaging (tumor‐to‐muscle ratio of 3.55 ± 1.38 after 90 min, Figure S5 and 4.32 ± 1.54 after 260 min, Figure 6D). Thus, in the TNBC model using human MDA‐MB‐231 cells, [^18^F]**1** has a much higher sensitivity than [^18^F]FDG in PET imaging.

### 2.9 Imaging of Pancreatic Cancer by [^18^F]1

**FIGURE 7:**
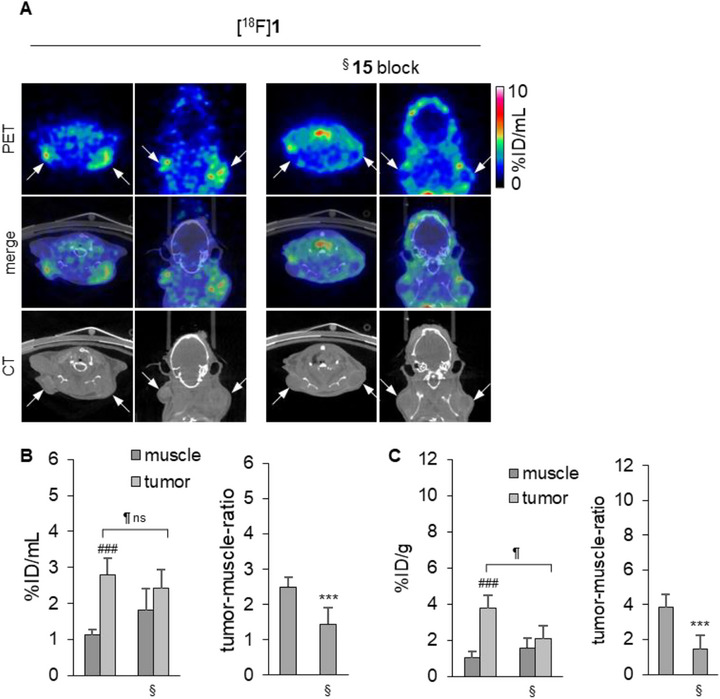
PET/CT imaging and quantitative analysis of [^18^F]**1** uptake in a pancreatic cancer cell (AsPC‐1) xenograft tumor model in mice. (A). PET images, CT scans, and merged images of [^18^F]**1**) 260 min after tracer injection. Left: no blocker. Right: Pre‐treatment with CD73 inhibitor **15** 10 min before tracer injection (§). White arrows mark the positions of tumors. (B). Quantitative PET analysis of [^18^F]**1** uptake: activity concentration of [^18^F]**1** in muscle and tumor tissues after 260 min without and with blocking (§) (left) and tumor‐to‐muscle ratios (right). [^18^F]**1**: *n* = 8 tumors, [^18^F]**1** plus compound **15** block: *n* = 8 tumors. (C). Ex vivo gamma counter analysis of explanted tissues: activity concentration of [^18^F]**1** in muscle and tumor tissues after 260 min without and with (§) blocking (left) and tumor‐to‐muscle ratios (right). [^18^F]**1**: *n* = 8 tumors, [^18^F]**1** plus compound **15** block: *n* = 8 tumors. ^#^relative to the respective muscle, *relative to [^18^F]**1** without blocker, relative to the unblocked tumor; *<0.05; **<0.01, ***<0.001, ^#^<0.05; ^##^ <0.01, ^###^<0.001,  <0.05;  <0.01,  <0.00, and ns: non‐significant, unpaired t‐test.

As previously mentioned, encouraging clinical results have been obtained with the nucleotide‐derived CD73 inhibitor Quemliclustat in the treatment of pancreatic cancer [48]. Therefore, we expanded our experiments to a human pancreatic cancer (AsPC‐1) mouse model. The AsPC‐1 cell line shows lower CD73 expression [49], is more aggressive and faster‐growing, and forms more diffuse tumors compared to the compact and well‐defined MDA‐MB‐231 tumors. In analogy to the results in the MDA‐MB‐231 tumor model, the pancreatic tumors accumulated the radiotracer [^18^F]**1**, while in the blocked mice, tumors could hardly be delineated on PET images ([^18^F]**1**, Figure 7A left). The tumor‐to‐muscle ratio was 2.49 ± 0.28, while it was reduced to 1.44 ± 0.47, after blocking (Figure 7B, C).

## 3 CONCLUSION

The development of small‐molecule ^18^F‐labeled CD73 inhibitors as novel PET tracers represents a significant advancement in the imaging of CD73 expression in cancer. So far, only a ^3^H‐labeled small‐molecule CD73 radioligand has been developed and utilized for in vitro radioligand binding assays and autoradiography studies [9]. Additionally, encouraging preclinical results were reported for an antibody‐based CD73‐targeting PET tracer [21]. While CD73 is ubiquitously expressed throughout the body, its upregulation is strongly correlated with tumor formation [50], and the prognostic value of CD73 expression was demonstrated in different cancer types, including diagnostically challenging TNBC and pancreatic cancer [15, 16, 51], but also on immune cells present in the TME or infiltrated into the tumor [52]. Motivated by the clinical need for a highly specific imaging tracer for such diagnostically challenging types of cancer, we developed two novel, highly potent, ^18^F‐labeled CD73 inhibitors, [^18^F]PSB‐19427 ([^18^F]**1,** K*_i _* = 1.02 ± 0.11 nM) and [^18^F]MRS‐4648 ([^18^F]**2,** K*_i_* = 0.664 ± 0.089 nM) as potential PET tracers for cancer imaging.

First, CD73 inhibitors **1** and **2** were evaluated in biopsy tissues of breast cancer patients, confirming the presence, activity, and tissue‐specific distribution of CD73. Subsequently, both compounds were prepared in radiolabeled form, starting from the respective precursors **7** or **12** via Huisgen cycloaddition using ^18^F‐labeled 2‐fluoroethyl azide, yielding [^18^F]**1** and [^18^F]**2** with satisfactory radiochemical yields and molar activities, and excellent rcp (>98%). Both tracers were stable when formulated for intravenous injection.

Since we observed that, tracers [^18^F]**1** and [^18^F]**2** bound selectively to CD73 in human breast cancer samples in autoradiography studies, we proceeded to biodistribution studies in female C57BL/6 WT mice. Despite a certain structural similarity, both radiotracers, being AMPCP (**13**) derivatives, the pyrimidine‐based [^18^F]**2** was rapidly excreted, while the purine‐based [^18^F]**1** displayed prolonged retention in blood and was present even after 260 min, indicating a good biodistribution and high metabolic stability in vivo. One possible explanation for the distinct in vivo behavior may be the compounds’ differences in PPB, 66% for **2** versus >99% for **1**, also reflected by the different radiosignal proportion in the blood (0.3% ± 0.1%ID/g for [^18^F]**2** versus 18.2% ± 1.7 %ID/g for [^18^F]**1** at 90 min p.i). High PPB is known to protect tracers from metabolic conversion, and increasing this parameter is employed as a common strategy to enhance the circulation time and bioavailability of radiotracers [53, 54]. Both radiotracers were metabolically rather stable in in vitro studies using MLM, compound **2** showing only 3% conversion within 90 min, while **1** was displaying 17% conversion. However, [^18^F]**2** was rapidly excreted in vivo, which prevented its accumulation in the MDA‐MB‐231 xenograft tumor model. In contrast, [^18^F]**1** accumulated in the tumor tissues, providing a tumor‐to‐muscle ratio of 4.32 ± 1.54 after 260 min. While tracer contrast (tumor‐to‐muscle ratio) was markedly reduced by applying non‐labeled **1** or the structurally distinct CD73 inhibitor **15**, the tumor signal was not significantly diminished by the blocking. A redistribution of the tracer into the muscle could be observed during the blocking experiments. This might again be explained by the high PPB of the compound, as the high blocker concentration (∼1000 fold higher than the radiotracer) displaced the tracer from the tumor tissue and allowed binding to the plasma protein, increasing the muscle signal. Furthermore, a saturation of the metabolically active enzymes and, thus, different metabolism and excretion, leading to slight differences in biodistribution, is feasible at this high blocker concentration. Beyond high affinity, the long residence time of **1** at CD73 may represent a key determinant of its in vivo imaging properties, and the incomplete blocking behavior observed for [^1^ ^8^F]**1** is consistent with reports on clinically translated immuno‐PET tracers such as ^8^ ^9^Zr‐pembrolizumab [44, 45] and ^1^ ^1^C‐l‐deprenyl [46], underscoring that kinetic and pharmacokinetic factors often dominate in vivo PET competition experiments. Moreover, altered drug distribution due to the pharmacological effects of the CD73 blocker might play a role since CD73 is expressed in blood vessels [3].

Next, we compared the performance of [^18^F]**1** in comparison to [^18^F]FDG in the MDA‐MB‐231 xenograft mouse model. While [^18^F]FDG remains a cornerstone of PET imaging, particularly for its ability to detect a wide range of malignancies, its limitations are well‐documented. For example, [^18^F]FDG uptake can be nonspecific due to its dependence on glucose metabolism, which is also elevated in inflammatory conditions and non‐cancerous tissues. The MDA‐MB‐231 tumors were only weakly detectable by [^18^F]FDG imaging with a tumor‐to‐muscle ratio of 2.04. In contrast, in mice imaged with the tracer [^18^F]**1**, the tumors were clearly visible with a tumor‐to‐muscle ratio of 4.32 (3.55 after 90 min *p.i*., Figure S2). Thus, [^18^F]**1** showed higher tumor‐to‐muscle contrast to [^18^F]FDG in PET imaging of breast cancer, at least in the employed TNBC mouse model. [^18^F]**1** is specifically targeting CD73, providing a more precise and reliable imaging modality for tumors in which CD73 is upregulated. This specificity could reduce false positive signals and improve the accuracy of tumor detection, particularly in early‐stage cancers or in tissues where [^18^F]FDG yields inconclusive results.

Next, in order to demonstrate its applicability for the imaging of different types of tumors, we employed [^18^F]**1** in PET imaging of a human pancreatic cancer (AsPC‐1) mouse model, using a cancer cell line known for its lower CD73 expression in comparison to MDA‐MB‐231 cells. Again, [^18^F]**1** displayed a pronounced tumor accumulation with a tumor‐to‐muscle ratio of 2.49 ± 0.28 after 260 min. Despite the lack of a pronounced blocking effect with the structurally different CD73 inhibitor **15**, multiple independent lines of evidence support that [^1^ ^8^F]**1** binds CD73 specifically and with high affinity. First, compound **1** is a potent inhibitor of human CD73 (K*_i_* = 2.78 nM; Table 1) and direct structural validation was obtained by an x‐ray co‐crystal structure of CD73 in complex with **1**, confirming active‐site binding and a well‐defined interaction mode (Figure 1B; PDB 9HD5). Second, specificity was corroborated at the tissue level: CD73 expression and enzymatic AMPase activity were mapped in human tumor sections (Figure 2), and incubation with **1** strongly reduced AMPase activity in situ (Figure 3), demonstrating functional target engagement in the same tissue context used for tracer evaluation. Finally, in situ autoradiography showed robust and displaceable binding of [^1^ ^8^F]**1** that was markedly reduced by excess CD73 inhibitors (AMPCP (**13**) or **14**; Figure 4).

CD73 upregulation is strongly correlated with the formation of solid tumors, and therefore, radiolabeled CD73 inhibitors have high potential as pan‐PET tracers for tumor imaging. High‐resolution methods for solid tumor imaging remain an unmet medical need and, if clinically successful, could save many lives through early cancer detection. The promising results with [^18^F]**1** suggest that CD73‐targeted PET imaging could play a crucial role in the diagnosis and staging of cancers with high CD73 expression. Furthermore, the ability of [^18^F]**1** to outperform [^18^F]FDG in specific contexts points to its potential as a preferred imaging agent in certain clinical scenarios, particularly for cancers that are difficult to detect with current methods. Due to CD73's functional relevance and potential as a therapeutic target in immuno‐oncology, the theranostic use of radiolabeled CD73 inhibitors appears promising [9].

This study is not without limitations. The radiolabeling of [^18^F]**1** through the Huisgen cycloaddition reaction relies on copper catalysis. Developing alternative radiolabeling strategies or modifying the tracer's chemical structure to enable copper‐free labeling could facilitate its transition to clinical settings. Additionally, while the current study focuses on breast and pancreatic cancers, expanding the evaluation of [^18^F]**1** to other cancer types where CD73 is overexpressed could further validate its utility as a pan‐cancer imaging agent.

The partial reduction in tumor uptake observed in in vivo blocking experiments is consistent with the tracer's pharmacokinetic properties, including high PPB and prolonged tissue retention; therefore, future studies would benefit from further optimizing the properties of the tracer and from including CD73‐negative tumor models as controls.

Moreover, due to the polar structure of the diphosphonate‐bearing nucleotide analog **1**, the developed PET tracer cannot cross the blood‐brain barrier and cannot be used for brain imaging unless administered intrathecally.

In conclusion, the development of [^18^F]**1** and [^18^F]**2** marks an important step forward in the field of cancer imaging. The high specificity and favorable biodistribution of [^18^F]**1**, in particular, suggest that it could become a valuable tool for the early detection and monitoring of cancers with elevated CD73 expression. Continued research toward the optimization and clinical translation of these tracers will be critical to realize their full potential for the imaging of solid tumors.

## Author Contributions

Conceptualization: Anna Junker, Christa E. Muller. Methodology: Anna Junker, Christa E. Muller. Investigation: Anna Junker, Clemens Dobelmann, Stefan Wagner, Lucie Grey, Jennifer Dabel, Andreas Isaak, Constanze C. Schmies, Georg W. Rolshoven, and Mirko Scortichini. Visualization: Sonja Schelhaas, Sandra Hoppner, and Gennady G. Yegutkin, Funding Acquisition: Anna Junker, Christa E. Muller. Project Administration: Anna Junker. Supervision: Anna Junker, Kenneth A. Jacobson, Christa E. Muller, and Sonja Schelhaas. Writing—Original Draft: Anna Junker, Christa E. Muller. Writing—Review & Editing: Anna Junker, Christa E. Muller, with support from all coauthors, who also reviewed and edited the manuscript.

## Funding

German Research Foundation (DFG) Emmy Noether program JU 2966/2‐2 (AJ), German Research Foundation (DFG) SFB 1328 and GRK 2873 (CEM), German Research Foundation (DFG)—EXC 2180 – 390900677 (AJ), NIH (NIDDK) Intramural grant ZIADK031117 (KAJ)

## Conflicts of Interest

The authors declare no conflicts of interest. Anna Junker, Kenneth A. Jacobson, and Christa E. Muller are coinventors on patent no. WO2020/037275A1.Anna Junker, Clemens Dobelmann, Christa E. Müller, Sonja Schelhaas, Sandra Höppner, and Stefan Wagner are coinventors on patent no. PCT/EP2023/054046.

## Supporting information




**Supporting File 1**: Detailed synthesis results, assay procedures, crystal structure analysis, stability in MLM, human serum albumin (HSA) binding, in vitro stability in mouse and human serum, tissue collection and preparation, immunofluorescence staining, in situ enzyme histochemistry, autoradiography, human tumor xenograft experiments, in vivo imaging, ex vivo gamma counter measurements, and NMR spectra of key intermediates, Figures S1–S6, Tables S1–S5 are provided within the Supporting Information. The authors have cited additional references within the Supporting Information.
**Supporting File 2**: anie71821‐sup‐0001‐SuppMat.docx.

## Data Availability

The data that support the findings of this study are available from the corresponding author upon reasonable request.
