# Supplementary material for: Fluorine-18-Labeled Nucleotide Analogs Targeting Ecto-5’-Nucleotidase (CD73) for Positron Emission Tomography Imaging of Solid Tumors
Source: Angew Chem Int Ed Engl. Author manuscript; Available in PMC 2026 Apr 15. (PMC13077603; doi:10.1002/anie.202522758)
Supplement: Supplementary file 1 [file NIHMS2157785-supplement-Supp_material_file.docx]

Supplementary Materials for

Fluorine-18-labeled Nucleotide Analogs Targeting Ecto-5'-nucleotidase (CD73) for Positron Emission Tomography Imaging of Solid Tumors

Clemens Dobelmann^1^†, Constanze C. Schmies^2^†, Georg Wilhelm Rolshoven^2^, Mirko Scortichini^3^, Stefan Wagner^4^, Andreas Isaak^1^, Riham M. Idris^2^, Jennifer Dabel^1^, Lucie Grey^5^, Karolina Losenkova^6^, Susanne Moschütz^9^, Haneen Al Hroub^2^, Antje Keim^9^, Sandra Höppner^1^, Jouko Sandholm^7^, Pia Boström^8^, Maija Hollmén^6^, Norbert Sträter^9^, Sven Hermann^1^, Gennady G. Yegutkin^6^, Kenneth A. Jacobson^3^, Sonja Schelhaas^1^, Christa E. Müller^2^*, Anna Junker^1, 10^*

^1 ^European Institute for Molecular Imaging (EIMI), University of Muenster; Roentgenstr 16, 48149 Münster, Germany. 

^2^ PharmaCenter Bonn, Pharmaceutical Institute, Pharmaceutical & Medicinal Chemistry University of Bonn; An der Immenburg 4, 53121 Bonn, Germany.

^3^ Molecular Recognition Section, Laboratory of Bioorganic Chemistry, National Institute of Diabetes and Digestive and Kidney Diseases, National Institutes of Health; Bethesda, MD 20892 USA.

^4 ^Department of Nuclear Medicine, University Hospital Muenster; Albert-Schweitzer-Campus 1, Building A1, 48149 Münster, Germany.

^5^ Institut für Pharmazeutische und Medizinische Chemie, University of Muenster; Corrensstr. 48, 48149 Muenster, Germany.

^6 ^Medicity Research Laboratory and InFLAMES Flagship, University of Turku; 20520 Turku, Finland.

^7 ^Turku Bioscience Centre, University of Turku and Åbo Akademi University; 20520 Turku, Finland.

^8^ Department of Pathology, University of Turku and Turku University Central Hospital; 20520 Turku, Finland.

^9 ^Institute of Bioanalytical Chemistry, Center for Biotechnology and Biomedicine, University of   Leipzig; Deutscher Platz 5, 04103 Leipzig, Germany.

^10^ Werner Siemens Imaging Center, Eberhard Karls University of Tuebingen, Department of Preclinical Imaging and Radiopharmacy; Cluster of Excellence iFIT (EXC 2180) “Image-guided and Functionally Instructed Tumor Therapies”, Roentgenweg 13, 72076 Tuebingen, Germany. 

† These authors contributed equally to this work.

*Corresponding author. Email: [christa.mueller@uni-bonn.de](mailto:christa.mueller@uni-bonn.de), anna.junker@med.uni-tuebingen.de 

**This PDF file includes:**

Fluorine-18-labeled Nucleotide Analogs Targeting Ecto-5'-nucleotidase (CD73) for Positron Emission Tomography Imaging of Solid Tumors	1

Fig S1: HPLC chromatograms of [^18^F]PSB-19427 ([^18^F]1). A): Rcp of [^18^F]1. B): [^18^F]1 spiked with non-labeled 1.	5

Fig S2: HPLC chromatograms of [^18^F]MRS-4648 ([^18^F]2). A: Rcp of [^18^F]2. B: [^18^F]2 spiked with non-labeled 2.	6

Fig S3: *In vitro* stability of the developed PET tracers in mouse and human serum.	7

Fig. S4: Dynamic tracer uptake of [^18^F]PSB-19427 ([^18^F]1) in two MDA-MB-231 tumor-bearing mice	9

Fig S5: Quantitative PET analysis of [^18^F]1 uptake: concentration of [^18^F]1 after 90 and 260 min expressed as tumor-to-muscle ratios.	9

Table S1: Details of crystallographic data collection and refinement	11

Table S2. QuPath v.0.3.0 scripts for staining intensity measurement.	12

Table S3: Tumor volumes at the point of imaging	13

Table S4: Selectivity of PSB-19427 (1) versus the human P2Y_1_ receptor	14

Table S5: Selectivity of PSB-19427 (1) versus the human P2Y_12_ receptor	14

MATERIALS AND METHODS	14

Study design	14

Experimental section	15

Chemistry- General	15

Preparation of triethylammonium hydrogencarbonate (TEAC) buffer	16

2,6-Dichloro-9-(2′,3′,5′-tri-*O*-acetyl-β-D-ribofuranosyl)-9*H*-purine (5)	16

(2*R*,3*R*,4*S*,5*R*)-2-{2-Chloro-6-[(4-ethynylbenzyl)(propyl)amino]-9*H*-purin-9-yl}-5-(hydroxymethyl)tetrahydrofuran-3,4-diol (6)	16

[((2*R*,3*S*,4*R*,5*R*)-5-{2-Chloro-6-[(4-ethynylbenzyl)(propyl)amino]-9*H*-purin-9-yl}-3,4-dihydroxytetrahydrofuran-2-yl)methoxy] (phosphonomethyl)-phosphonic acid (7)	17

{[{[(2*R*,3*S*,4*R*,5*R*)-5-(2-Chloro-6-{4-[1-(2-fluoroethyl)-1*H*-1,2,3-triazol-4-yl) benzyl](propyl)amino}-9*H*-purin-9-yl)-3,4-dihydroxytetrahydrofuran-2-yl]methoxy{(hydroxy)phosphoryl]methyl}phosphonic acid (1, PSB-19427).	17

1-[(2*R*,3*R*,4*S*,5*R*)-3,4-Dihydroxy-5-(hydroxymethyl)tetrahydrofuran-2-yl]-4-{[(4-ethynylbenzyl)oxy]imino}-3-methyl-3,4-dihydropyrimidin-2(1*H*)-one (9)	18

(2*R*,3*R*,4*R*,5*R*)-2-(Acetoxymethyl)-5-(4-{[(4-ethynylbenzyl)oxy]imino}-3-methyl-2-oxo-3,4-dihydropyrimidin-1(2*H*)-yl)tetrahydrofuran-3,4-diyl diacetate (10)	19

1-[(2*R*,3*R*,4*S*,5*R*)-3,4-Dihydroxy-5-(hydroxymethyl)tetrahydrofuran-2-yl]-4-[({4-[1-(2-fluoroethyl)-1*H*-1,2,3-triazol-4-yl]benzyl}oxy)imino]-3-methyl-3,4-dihydropyrimidin-2(1*H*)-one (11)	20

{[{[(2*R*,3*S*,4*R*,5*R*)-5-(-4-{[(4-Ethynylbenzyl)oxy]imino}-3-methyl-2-oxo-3,4-dihydropyrimidin-1(2*H*)-yl)-3,4-dihydroxytetrahydrofuran-2-yl]methoxy}(hydroxy)phosphoryl]methyl}phosphonic acid (12)	21

[((2*R*,3*S*,4*R*,5*R*)-5-{4-[({4-[1-(2-Fluoroethyl)-1*H*-1,2,3-triazol-4-yl]benzyl}oxy)-imino]-3-methyl-2-oxo-3,4-dihydropyrimidin-1(2*H*)-yl}-3,4-dihydroxytetrahydro-furan-2-yl)methoxy]-(phosphonomethyl)phosphonic acid (MRS-4648 (2))	22

Radiochemistry. General Methods.	23

[^18^F]-[((2*R*,3*S*,4*R*,5*R*)-5-{2-chloro-6-[(4-{1-[2-(fluoro)ethyl]-1*H*-1,2,3-triazol-4-yl}benzyl)-(propyl)amino]-9*H*-purin-9-yl}-3,4-dihydroxytetrahydrofuran-2-yl)methoxy]methylene-bisphosphonic acid ([^18^F]PSB-19427, [^18^F]1)	24

[^18^F]-{[(2*R*,3*S*,4*R*,5*R*)-5-(4-{[(4-{1-[2-(fluoro)ethyl]-1*H*-1,2,3-triazol-4-yl}benzyl)-oxy]imino}-3-methyl-2-oxo-3,4-dihydropyrimidin-1(2*H*)-yl)-3,4-dihydroxytetra-hydrofuran-2-yl]methoxy}methylenebisphosphonic acid ([^18^F]MRS-4648, [^18^F]2)	24

Crystal structure analysis	25

Determination of the logD_7.4_–values of radiotracers	26

Stability in mouse liver microsomes (MLM)	26

Human serum albumin (HSA) binding.	26

*In vitro* stability in mouse and human serum	27

Enzyme inhibition assay	27

Tissue collection and preparation	27

Immunofluorescence staining	27

*In situ* enzyme histochemistry	28

Autoradiography	28

Human tumor xenograft experiments	29

In vivo imaging	29

Ex vivo gamma counter measurements	30

Determination of kinetic rate index and estimation of residence time	30

NMR data analysis	30

Statistical analysis	30

NMR spectra, HPLC purity, HRMS spectra, FT-IR spectra of selected key intermediates	1

(2*R*,3*R*,4*S*,5*R*)-2-{2-Chloro-6-[(4-ethynylbenzyl)(propyl)amino]-9*H*-purin-9-yl}-5-(hydroxymethyl)tetrahydrofuran-3,4-diol (6)	1

[((2*R*,3*S*,4*R*,5*R*)-5-{2-Chloro-6-[(4-ethynylbenzyl)(propyl)amino]-9*H*-purin-9-yl}-3,4-dihydroxytetrahydrofuran-2-yl)methoxy] (phosphonomethyl)-phosphonic acid (7)	3

{[{[(2*R*,3*S*,4*R*,5*R*)-5-(2-Chloro-6-{4-[1-(2-fluoroethyl)-1*H*-1,2,3-triazol-4-yl) benzyl](propyl)amino}-9*H*-purin-9-yl)-3,4-dihydroxytetrahydrofuran-2-yl]methoxy{(hydroxy)phosphoryl]methyl}phosphonic acid (1, PSB-19427).	6

1-[(2*R*,3*R*,4*S*,5*R*)-3,4-Dihydroxy-5-(hydroxymethyl)tetrahydrofuran-2-yl]-4-[({4-[1-(2-fluoroethyl)-1*H*-1,2,3-triazol-4-yl]benzyl}oxy)imino]-3-methyl-3,4-dihydropyrimidin-2(1*H*)-one (11)	9

{[{[(2R,3S,4R,5R) 5 ( 4 {[(4 Ethynylbenzyl)oxy]imino} 3 methyl 2 oxo 3,4-dihydropyrimidin 1(2H) yl) 3,4 dihydroxytetrahydrofuran 2-yl]methoxy}(hydroxy)phosphoryl]methyl}phosphonic acid (12)	18

[((2*R*,3*S*,4*R*,5*R*)-5-{4-[({4-[1-(2-Fluoroethyl)-1*H*-1,2,3-triazol-4-yl]benzyl}oxy)-imino]-3-methyl-2-oxo-3,4-dihydropyrimidin-1(2*H*)-yl}-3,4-dihydroxytetrahydro-furan-2-yl)methoxy]-(phosphonomethyl)phosphonic acid (MRS-4648 (2))	26

References	36

# Fig S1: HPLC chromatograms of [^18^F]PSB-19427 ([^18^F]1). A): Rcp of [^18^F]1. B): [^18^F]1 spiked with non-labeled 1.

|  |
| --- |
|  |

# Fig S2: HPLC chromatograms of [^18^F]MRS-4648 ([^18^F]2). A: Rcp of [^18^F]2. B: [^18^F]2 spiked with non-labeled 2.

The shoulder observed in the HPLC chromatogram of compound **2** resulted from a temporary issue with the HPLC system rather than from sample heterogeneity. This was confirmed by the fact that compound **1**, analyzed under the same conditions at that time, exhibited a similar shoulder. After the instrument issue was identified and resolved, compound **1** was re-analyzed, and no shoulder was observed, demonstrating that the phenomenon was equipment-related. The radiolabeling experiment for compound **2** was not repeated solely for the purpose of acquiring a new chromatogram; therefore, the originally recorded chromatogram is shown, despite the transient system-related artifact. Importantly, this does not affect the conclusions drawn from the radiolabeling experiment.

# Fig S3: *In vitro* stability of the developed PET tracers in mouse and human serum.

Mouse serum stability of [^18^F]MRS-4648.

Human serum stability of [^18^F]MRS-4648.

Mouse serum stability of [^18^F]PSB-19427.

Human serum stability of [^18^F]PSB-19427.

# Fig. S4: Dynamic tracer uptake of [^18^F]PSB-19427 ([^18^F]1) in two MDA-MB-231 tumor-bearing mice

# Fig S5: Quantitative PET analysis of [^18^F]1 uptake: concentration of [^18^F]1 after 90 and 260 min expressed as tumor-to-muscle ratios. 

**Fig S6: Comparison of inhibitor binding modes.** A) Polder omit electron density of JMS04-14 contoured at 5 σrms. B) Polder omit electron density map of PSB12489 contoured at 4.0 σrms (blue) and at 3.0 σrms (gray). C) Superposition of the binding modes of PSB19427, PSB12489 (pdb id 6s7h) and AOPCP (4h2i). The two red arrows mark the movements of the N6 atom of the inhibitors and of the region 184-196. The structures have been superimposed based on the Cα atoms of the C-terminal domain. D) Side view of the superposition of JMS04-14 and PSB19427 based on residues of both protein domains.

# Table S1: Details of crystallographic data collection and refinement

| PDB code | **9HD5 (PSB19427)** | **9SME (JMS04-14)** |
| --- | --- | --- |
| **Crystallization and data collection** |  |  |
| Crystallization buffer | 18% PEG 6000; 0.1 M BisTrisPropane pH 7.0, 10 µM ZnCl_2_ | 9 % PEG 6.000; 0,1 M BisTrisPropane pH 5.7 |
| Crystallization drop | 1 µL crystallization buffer + 1 µL of 7 mg/mL CD73 in 10 mM Tris pH 8.0, 10 µM ZnCl_2, _1 mM PSB19427 | 1 µL crystallization buffer + 1 µL of 7 mg/mL CD73 in 10 mM Tris pH 8.0, 10 µM ZnCl_2_, 10 mM JMS04-14 |
| Cryobuffer | cryst. buffer + 20 % PEG200 | crystallization buffer + 20 % PEG200, 10 µM ZnCl_2_, 10 mM JMS04-14 |
| Wavelength (Å) | 0.91841 | 0.97932 |
| Resolution range (Å)^1^ | 49.37-2.91 (3.08-2.91) | 94.38-2.55 (2.65-2.55) |
| Space group | P 2_1_ 2_1_ 2 | P 2_1_ 2_1_ 2 |
| Unit cell parameters a, b, c (Å) | 92.63 233.36 54.06 | 94.38 235.39 53.80 |
| Unique reflections | 16993 (849) | 40069 (4425) |
| Multiplicity | 12.5 (11.3) | 8.0 (8.3) |
| Completeness (%) | 90.8 (65.4) | 99.8 (99.6) |
| Mean I/sigma(I) | 5.5 (1.1) | 7.5 (0.9) |
| R-meas | 0.992 (8.493) | 0.112 (2.140) |
| R-pim | 0.279 (2.606) | 0.039 (0.738) |
| R-merge | 0.951 (8.048) | 0.105 (2.006) |
| CC1/2 | 0.977 (0.122) | 0.999 (0.836) |
| Resolution aniso (Å)* | 4.38, 2.81, 2.85 | 2.87, 2.55, 2.55 |
| Wilson B-factor (Å^2^) | 56.9 | 69.3 |
| **Refinement** |  |  |
| Resolution range (Å) | 49.37-2.911 (2.99-2.91) | 87.60-2.550 (2.57-2.55) |
| R-work | 0.1896 (0.2488) | 0.2546 (0.2657) |
| R-free | 0.2725 (0.4649) | 0.2878 (0.2475) |
| Mean B-factor (Å^2^) | 57.7 | 57.7 |
| Number of non-hydrogen atoms |  |  |
| Protein | 8005 | 8016 |
| Heterogen (ligands, metals) | 96 | 70 |
| solvent | 118 | 144 |
| Root mean square deviations |  |  |
| Bonds (Å) | 0.008 | 0.008 |
| Angles (°) | 1.06 | 1.05 |
| Ramachandran statistics (Molprobity) |  |  |
| Favored (%) | 93.12 | 94.81 |
| Allowed (%) | 6.0 | 4.90 |
| Outliers (%) | 0.88 | 0.29 |

*Diffraction limits along a*, b* and c* as determined by the Staraniso algorithm

# Table S2. QuPath v.0.3.0 scripts for staining intensity measurement.

| A runPlugin('qupath.imagej.detect.tissue.SimpleTissueDetection2', '{"threshold": 254, "requestedPixelSizeMicrons": 20.0, "minAreaMicrons": 2000000.0, "maxHoleAreaMicrons": 1000000.0, "darkBackground": false, "smoothImage": true, "medianCleanup": true, "dilateBoundaries": false, "smoothCoordinates": true, "excludeOnBoundary": true, "singleAnnotation": false}'); |
| --- |
| B setColorDeconvolutionStains('{"Name" : "H-DAB default", "Stain 1" : "Hematoxylin", "Values 1" :"0.65111 0.70119 0.29049 ", "Stain 2" : "DAB", "Values 2" : "0.26917 0.56824 0.77759 ", "Background" :" 255 255 255 "}'); |
| C runPlugin('qupath.lib.algorithms.IntensityFeaturesPlugin', '{"pixelSizeMicrons": 2.0,  "region": "ROI",  "tileSizeMicrons": 25.0,  "colorOD": false,  "colorStain1": false,  "colorStain2": true,  "colorStain3": false,  "colorRed": false,  "colorGreen": false,  "colorBlue": false,  "colorHue": false,  "colorSaturation": false,  "colorBrightness": false,  "doMean": true,  "doStdDev": false,  "doMinMax": false,  "doMedian": false,  "doHaralick": false,  "haralickDistance": 1,  "haralickBins": 32}'); |

(A) Whole tissue areas were detected with the Simple Tissue Detection function. (B) The default DAB color deconvolution values were used as a proxy for AMPase activity (brown deposit) analysis. (C) The intensities on the DAB channel were measured from the whole tissue areas using the Intensity Features function. 

# Table S3: Tumor volumes at the point of imaging

| ID | Right tumor volume (mm^3^) | Left tumor volume (mm^3^) | Tumor Entity |  | Mean Volume | SD |
| --- | --- | --- | --- | --- | --- | --- |
| 13805 | 319.4 | 414.3 | MDA-MB-231 | MDA-MB-231 | 129.7 | 136.6 |
| 13807 | 130.2 | 185.7 | MDA-MB-231 | As-PC-1 | 69.0 | 20.9 |
| 13809 | 632.2 | 289.0 | MDA-MB-231 |  |  |  |
| 13953 | 160.7 | 88.7 | MDA-MB-231 |  |  |  |
| 13954 | 143.4 | 141.5 | MDA-MB-231 |  |  |  |
| 14086 | 21.4 | 32.5 | MDA-MB-231 |  |  |  |
| 14087 | 29.7 | 27.4 | MDA-MB-231 |  |  |  |
| 14089 | 100.7 | 37.0 | MDA-MB-231 |  |  |  |
| 14090 | 48.1 | 57.1 | MDA-MB-231 |  |  |  |
| 14092 | 51.8 | 59.2 | MDA-MB-231 |  |  |  |
| 14340 | 102.3 | 80.0 | MDA-MB-231 |  |  |  |
| 14341 | 165.5 | 93.8 | MDA-MB-231 |  |  |  |
| 14342 | 85.7 | 40.8 | MDA-MB-231 |  |  |  |
| 14343 | 42.9 | 47.6 | MDA-MB-231 |  |  |  |
| 14344 | 50.6 | 64.5 | MDA-MB-231 |  |  |  |
| 14345 | 46.6 | 115.2 | MDA-MB-231 |  |  |  |
| 14346 | 145.8 | 47.4 | MDA-MB-231 |  |  |  |
| 14347 | 83.2 | 120.8 | MDA-MB-231 |  |  |  |
| 14348 | 150.0 | 106.0 | MDA-MB-231 |  |  |  |
| 14349 | 83.8 | 94.1 | MDA-MB-231 |  |  |  |
| 13971 | 69.6 | 84.7 | As-PC-1 |  |  |  |
| 13972 | 78.0 | 37.6 | As-PC-1 |  |  |  |
| 13974 | 66.0 | 49.4 | As-PC-1 |  |  |  |
| 13975 | 91.9 | 102.3 | As-PC-1 |  |  |  |
| 13976 | 101.9 | 60.7 | As-PC-1 |  |  |  |
| 14084 | 55.7 | 47.4 | As-PC-1 |  |  |  |
| 14093 | 67.3 | 55.7 | As-PC-1 |  |  |  |
| 14094 | 30.0 | 55.0 | As-PC-1 |  |  |  |
| 14095 | 60.3 | 58.8 | As-PC-1 |  |  |  |

Table S4: Selectivity of PSB-19427 (1) versus the human P2Y_1_ receptor

| **Compound** | **Inhibition/Activation of the human P2Y**_**1**_ **receptor**^a^ |  |
| --- | --- | --- |
|  | **at 1 µM (%) ± SEM** | **at 10 µM (%) ± SEM** |
| *as antagonist*^*b*^ |  |  |
| PSB-19427 (**1**) | **-12** ± 3 | **16** ± 6 |
| *as agonist* |  |  |
| PSB-19427 (**1**) | **7** ± 1 | **34** ± 6 |

^**a**^Determined in calcium mobilization assays in 1321N1 astrocytoma cells recombinantly expressing the human P2Y_1_ receptor.^[54]^

^**b**^ADP was used as a standard agonist at a concentration corresponding to its EC_80_.

Table S5: Selectivity of PSB-19427 (1) versus the human P2Y_12_ receptor

**Table 3:** Screening results of PSB-19427 at the hP2Y_12_ receptor as agonists and antagonists in as determined by β-arrestin assay. The receptor was expressed in CHO-PK1 cells.

| **Compound** | **Inhibition/Activation of the human P2Y**_**12**_ **receptor**^a^ |  |
| --- | --- | --- |
|  | **at 1 µM (%) ± SEM** | **at 10 µM (%) ± SEM** |
| *as antagonist*^*b*^ |  |  |
| PSB-19427 (**1**) | **28** ± 2 | **19** ± 2 |
| *as agonist* |  |  |
| PSB-19427 (**1**) | **-3** ± 7 | **-9** ± 13 |

^a^Determined in ß-arrestin recruitment assays employing ß-galactosidase complementation in Chinese hamster ovary CHO-K1 cells recombinantly expressing the human P2Y_12 _receptor.^[54]^

^b^2-MeSADP used as a standard agonist at the concentration of its corresponding EC_80._

MATERIALS AND METHODS

Study design

The primary research objective was to design and synthesize an imaging agent to image tumor-specific CD73 expression in living subjects noninvasively via PET. Six to ten animals per group were used to evaluate CD73 expression in xenograft models of breast (MDA-MB-231) and pancreatic (AsPC-1) cancer. All outliers were included in the analysis, and no data were excluded. The authors were not blinded to the results. A minimum of three experimental replicates were recorded for all in vitro data.

Adult, 9 to 13 week old, C57bl/6 (biodistribution study, 19.8±1.3 g) or 7 to 12 weeks old NOD.Cg-*Prkdc*^*scid*^ *Il2rg*^*tm1Wjl*^/SzJ mice (22.3±2.2 g) were obtained from Charles River Laboratories. A total of 43 mice were used for this work

All animal experiments were performed in accordance with the legal requirements of the European Community (Directive 2010/63/EU) and the corresponding German Animal Welfare Law (TierSchG, TierSchVersV) and were approved by the local authorizing agency (State Office for Nature, Environment and Consumer Protection North Rhine-Westphalia).

Experimental section

Chemistry- General 

All reagents were commercially obtained from various suppliers (Alfa Aesar, Carbosynth, and Sigma Aldrich) and used without further purification. The purity of all compounds, including the starting materials, was greater than 95%, as determined by HPLC. Commercial solvents of specific reagent grades were used without additional purification or drying. Analytical thin-layer chromatography was carried out on Sigma-Aldrich® TLC plates and compounds were visualized with UV light at 254 nm. Flash column chromatography (fc): Silica gel 60, 40–64 µm; parentheses include: diameter of the column, length of the column, fraction size, eluent, R_f_ value. Melting point: melting point apparatus Stuart Scientific® SMP 3, uncorrected. IR: IR spectrophotometer FT-ATR-IR (Jasco®). The ^1^H, ^31^P, and ^13^C NMR spectra were recorded using Bruker 400 MHz spectrometer, a DD2 400 MHz or DD2 600 MHz NMR spectrometer (Agilent). DMSO-d_6_, MeOD-d_4_, CDCl_3_ or D_2_O were used as solvents. Shifts are given in ppm relative to the remaining protons of the deuterated solvents used as internal standard (^1^H-, ^13^C-NMR) or using D_3_PO_4_ as external standard (^31^P), the assignments of ^13^C and ^1^H NMR signals were supported by 2D NMR techniques. Purification of final compounds was performed by semi-preparative HPLC (Thermo Fisher Scientific,  Ultimate™ 3000; Column: Luna 5 µm C18(2) 100 Å, LC Column 250 x 4.6 mm). Eluent: 10 mM triethylammonium acetate buffer - CH_3_CN from 80:20 to 20:80 in 40 min, with a flow rate of 5 mL/min. Purities of all tested compounds were ≥95%, as estimated by analytical HPLC: Equipment: UV-detector: UltiMate 3000 variable Wavelength Detector; autosampler: UltiMate 3000; pump: Ultimate 3000; degasser: Ultimate 3000: data acquisition: Chromeleon Client 8.0.0 (Dionex Corpor.). Method: flow rate: 1.00 mL/min; injection volume: 5.0 µL; Method A: Eluent: 5 mM triethylammonium phosphate monobasic solution - CH_3_CN from 100:0 to 50:50 in 20 min, then triethylammonium phosphate monobasic solution - CH_3_CN to 100:0 in 5 min with a flow rate of 1 mL/min (Column: Zorbax SB-Aq 5 µm, 80 Å, 4.6 X 50 mm; Agilent Technologies, Inc). Method B: Eluent: 5 mM triethylammonium phosphate monobasic solution - CH_3_CN from 90:10 to 0:100 in 20 min, then triethylammonium phosphate monobasic solution - CH_3_CN from 0:100 to 90:10 in 5 min with a flow rate of 1 mL/min (Column: Zorbax SB-Aq 5 µm, 80 Å, 4.6 X 150 mm; Agilent Technologies, Inc). Method C: Eluent: 5 mM triethylammonium phosphate monobasic solution - CH_3_CN from 80:20 to 20:80 in 20 min, then triethylammonium phosphate monobasic solution - CH_3_CN from 20:80 to 80:20 in 10 min with a flow rate of 1 mL/min (Column: Zorbax SB-Aq 5 µm analytical column, 150 X 4.6 mm; Agilent Technologies, Inc). Peaks were detected by UV absorption (210 nm) using a UV/VIS detector. Low-resolution mass spectrometry was performed with a JEOL SX102 spectrometer with 6-kV Xe atoms following desorption from a glycerol matrix or on an Agilent LC/MS 1100 MSD, with a Waters (Milford, MA) Atlantis C18 column. High-resolution mass spectroscopic (HRMS) measurements were performed on a proteomics optimized Q-TOF-2 (Micromass-Waters) using external calibration with polyalanine or a MicroTof (Bruker Daltronics, Bremen), calibration with sodium formate clusters before measurement. For lyophilization, a freeze dryer (Labconco FreeZone 4.5) was used.

Preparation of triethylammonium hydrogencarbonate (TEAC) buffer

A 1 M solution of TEAC was prepared by the following procedure: to a 1 M solution of triethylamine in deoinized water was added dry ice slowly until the pH value reached approximately 8.4-8.6. 

2,6-Dichloro-9-(2′,3′,5′-tri-*O*-acetyl-β-D-ribofuranosyl)-9*H*-purine (5)

1,2,3,5-Tetraacetyl-β-D-ribofuranose (**4**, 5 g, 16.0 mmol, 1.0 eq) was molten at 85 °C, and 2,6-dichloropurine (**3**, 3.0 g, 16.0 mmol, 1.0 eq) was added while stirring. Trifluoromethanesulfonic acid (70 µL, 0.8 mmol, 0.05 eq) was added to the reaction mixture. The reaction mixture was stirred at 85 °C under reduced pressure for 30 min. Analysis by thin-layer chromatography (TLC) was performed to indicate the completion of the reaction. The mixture was then cooled to rt to allow crystallization. The formed solid was filtered off. Recrystallization from absolute ethanol yielded the desired product (4.84 g, yield 69%). ^1^H NMR (600 MHz, DMSO-*d*_*6*_) *δ* 8.90 (s, 1H, 8-CH), 6.30 (d, 1H, *J* = 4.9 Hz, 1ʹ-CH), 5.89 (q, 1H, *J* = 5.4 Hz, 2ʹ-CH), 5.61 (t, 1H, *J* = 5.6 Hz, 3ʹ-CH), 4.43-4.28 (m, 2H, 5ʹ-CH_2_), 4.38 (dd, 1 H, *J* = 3.6, 12.1 Hz, 4ʹ-CH), 2.10 (s, 3H, CH_3_), 2.04 (s, 3H, CH_3_), 2.00 (s, 3H, CH_3_). ^13^C NMR (126 MHz, DMSO-*d*_*6*_) *δ* 170.21, 169.55, 169.41, 152.96, 151.52, 150.47, 147.04, 13.43, 86.44, 80.03, 72.55, 69.94, 62.79, 20.65, 20.52, 20.38. LC-MS (*m/z*): positive mode 447.0 [M+H]^+^. Purity determined by HPLC-UV (254 nm)-ESI-MS: 88%. mp: 160 °C.

(2*R*,3*R*,4*S*,5*R*)-2-{2-Chloro-6-[(4-ethynylbenzyl)(propyl)amino]-9*H*-purin-9-yl}-5-(hydroxymethyl)tetrahydrofuran-3,4-diol (6)

2ʹ,3ʹ,5ʹ-Tri-*O*-acetyl-2,6-dichlororibofuranosylpurine (**5**, 0.34 g, 0.77 mmol, 1.0 eq) was suspended in absolute ethanol. Triethylamine (0.2 mL, 1.54 mol, 2.0 eq) and *N*-(4-ethynylbenzyl)propan-1-amine (0.26 g, 1.54 mmol, 2.0 eq) were added to the suspension that was refluxed overnight. The progress of the reaction was monitored by TLC (DCM/methanol 9/1). After TLC indicated the completion of the reaction, the solvent was evaporated. The subsequent deprotection reaction was carried out using 1 M sodium methoxide in methanol (10 mL). Purification by column chromatography (methanol/DCM 1:19) yielded the desired product as a white solid (0.25 g, 71%). ^1^H NMR (600 MHz, DMSO-*d*_*6*_) *δ* 8.42 (br s, 1H, 8-CH), 7.42 (d, 2H, *J* = 8.04 Hz, CH_benzene_), 7.28 (br s, 2H, CH_benzene_), 5.85 (d, *J* = 5.83 Hz, 1H, 1ʹ-CH), 5.54 (d, 1H, *J* = 12.20 Hz, N-CH_2_), 5.45 (br s, 1H, OH), 5.18 (br s, 1H, OH), 5.00 (br s, 1H, OH), 4.92 (d, 1H, *J* = 15.05 Hz, NCH_2_), 4.51 (br s, 1H, 2ʹ-CH), 4.12 (s, 2H, NCH_2_), 4.05 (q, 1H, *J* = 5.19 Hz, 3ʹ-CH), 3.94 (q, 1H, *J* = 3.92 Hz, 4ʹ-CH), 3.59 (dm, 2H, 5ʹ-CH_2_), 1.63 (br s, 2H, CH_2_CH_3_), 1.05 (t, 1H, *J* = 7.02 Hz, CCH), 0.85 (t, 3H, CH_3_). ^13^C NMR (126 MHz, CD_3_OD) *δ* 154.38, 152.79, 151.73, 139.27, 132.03, 127.78, 120.67, 118.46, 87.47, 85.85, 83.46, 80.83, 73.84, 70.48, 61.45, 39.72, 21.88, 18.60, 11.08. LC-MS (m/z): positive mode 458.2 [M+H]^+^. Purity determined by HPLC-UV (254 nm)-ESI-MS: 88%. mp. 110 °C.

[((2*R*,3*S*,4*R*,5*R*)-5-{2-Chloro-6-[(4-ethynylbenzyl)(propyl)amino]-9*H*-purin-9-yl}-3,4-dihydroxytetrahydrofuran-2-yl)methoxy] (phosphonomethyl)-phosphonic acid (7)

(2*R*,3*R*,4*S*,5*R*)-2-(2-Chloro-6-((4-ethynylbenzyl)(propyl)amino)-9*H*-purin-9-yl)-5-(hydroxymethyl)tetrahydrofuran-3,4-diol (**6**, 0.1 g, 0.22 mmol, 1.0 eq.) was dissolved in trimethyl phosphate (2 mL) and stirred at 0-4 °C. A solution of methylenebis(phosphonic dichloride) (0.27 g, 1.09 mmol. 5.0 eq.) in trimethyl phosphate (3 mL), cooled to 0-4 °C was added. The reaction mixture was stirred at 0-4 °C and samples were withdrawn at 15 min intervals for TLC to control for the disappearance of nucleosides. After 30 min, when the nucleoside had completely reacted, 20 mL of cold 0.5 M aqueous TEAC solution (pH 7.4-7.6) was added and the solution was stirred at 0 °C for 15 min followed by stirring at room temperature for 1 h. Trimethyl phosphate was extracted using 2 x 250 mL of *tert*-butyl methyl ether, and the aqueous layer was lyophilized. The crude product was then purified by RP-HPLC (0-50% CH_3_CN/50 mM NH_4_HCO_3_ buffer within 20 min, 20 mL/min) and the appropriate fractions were pooled. The product was obtained as a white solid after lyophilization (0.11 g, 89%). Purity determined by HPLC-UV (254 nm)-ESI-MS: 84%. mp: 141 °C.  ^1^H NMR (600 MHz, D_2_O) *δ* 8.37 (s, 1H, 8-C*H*), 7.30 (br s, 2H, 3-CH_phenyl_, 5-CH_phenyl_), 7.18 (d, 2H, *J* = 7.78 Hz, 2-CH_phenyl_, 6-CH_phenyl_), 6.01 (d, 1H, *J* = 5.36 Hz, 1ʹ-C*H*), 5.31 (br s, 2H, NC*H*_*2*_), 4.68 (t, 1H, *J* = 5.20 Hz, 2ʹ-C*H*), 4.51 (d, 1H, *J* = 4.70 Hz, 3ʹ-C*H*), 4.36 (d, 1H,  *J* = 3.79 Hz, 4ʹ-C*H*), 4.16 (t, 2H, *J* = 4.24 Hz, 5ʹ-C*H*_2_), 3.92 (br s, 2H, NC*H*_*2*_), 3.43 (s, 1H, CC*H*), 2.19 (t, 2H, *J* = 19.68 Hz, PC*H*_*2*_P), 1.62 (br s, 2H, C*H*_*2*_CH_3_), 0.84 (t, 3H, *J* = 7.55 Hz, C*H*_*3*_). ^13^C NMR (126 MHz, D_2_O) *δ* 167.5, 157.6, 156.4, 154.1, 141.3, 141.1, 134.9, 130.3, 130.1, 125.7, 123.1, 120.8, 89.8, 86.6, 85.9, 81.0, 77.2, 73.0, 66.4, 53.5, 30.4, 23.86, 13.1. ^31^P NMR (202 MHz, D_2_O) *δ* 18.95 (d, 1P, *J* = 9.50 Hz, β-*P*) 14.95 (d, 1P, *J* = 9.28 Hz, α-*P*). Exact mass (ESI): m/z = calcd. for C_23_H_29_ClN_5_O_9_P_2_ 616.1129, found 616.1130 [M+H]^+^ and calcd. for C_23_H_27_ClN_5_O_9_P_2 _614.0973, found 614.0991 [M-H]^-^. 

 {[{[(2*R*,3*S*,4*R*,5*R*)-5-(2-Chloro-6-{4-[1-(2-fluoroethyl)-1*H*-1,2,3-triazol-4-yl) benzyl](propyl)amino}-9*H*-purin-9-yl)-3,4-dihydroxytetrahydrofuran-2-yl]methoxy{(hydroxy)phosphoryl]methyl}phosphonic acid (1, PSB-19427). 

Compound **7** (15.0 mg, 0.02 mmol, 1.0 eq) was dissolved in THF/H_2_O/*t*-BuOH (3:1:1, 0.5 mL). Crude 2-fluoroethylazide 10 (50.0 μL) was added. Additionally, 1 M sodium ascorbate in H_2_O (0.03 mL, 0.03 mmol, 1.2 eq) was added. Finally, a premixed solution of CuSO_4_ (1.0 mg, 0.009 mmol, 0.3 eq) and TBTA (4.0 mg, 0.003 mmol, 0.3 eq) in THF/H_2_O/*t*-BuOH (3:1:1, 0.5 mL) was added to the reaction mixture. The reaction was stirred in the dark under argon at rt. After one night of stirring, the reaction mixture was evaporated and directly purified by RP-HPLC (0-70% CH_3_CN/50mM NH_4_HCO_3_ buffer within 20 min, 20 mL/min). Appropriate fractions were pooled and lyophilized overnight to obtain the final product as a white solid (0.003 g, 17.5%). Purity determined by HPLC-UV (254 nm)-ESI-MS: 99%. mp. 169 °C. ^1^H NMR (600 MHz, D_2_O) *δ* 8.40 (s, 1H, C*H*_triazole_), 8.27 (s, 1H, 8-C*H*_purine_), 7.67 (d, 2H, *J* = 6.91 Hz, 3-C*H*_phenyl_, 5-C*H*_phenyl_,), 7.36 (d, 2H, *J* = 7.90 Hz, 2-CH_phenyl_, 6-CH_phenyl_), 6.02 (d, 1H, *J* = 4.74 Hz, 1ʹ-C*H*), 4.93-4.85 (m, 2H, NC*H*_*2*_), 4.77 (m, 2H, overlapping with H_2_O: NC*H*_2_), 4.73 (s, 1H, 2ʹ-C*H*), 4.53 (s, 1H, 3ʹ-C*H*), 4.38 (s, 1H, 4ʹ-C*H*), 4.18 (br s, 2H, 5ʹ-C*H*_2_), 3.15 (m, 2H, NC*H*_2_), 2.18 (br s, 2H, PCH_2_P), 1.67 (m, 2H, C*H*_2_F), 1.34 (m, 2H, C*H*_*2*_CH_3_), 0.91 (m, 3H, C*H*_*3*_). ^13^C NMR (126 MHz, D_2_O) *δ* 159.1, 156.5, 154.1, 152.6, 143.9, 141.0, 131.3, 131.0, 128.6, 125.4, 120.8, 89.7, 86.7, 85.5, 77.1, 73.1, 66.5, 55.5, 53.8, 53.6, 49.5, 28.1, 15.6. ^31^P NMR (243 MHz, D_2 _O) δ 19.43, 14.48. ^19^F NMR (471 MHz, D_2_O) δ -223.05.Exact mass (ESI): m/z = calcd. for C_25_H_33_ClFN_8_O_9_P_2_ 705.1518, found 705.1486 [M+H]^+^ and calcd. for C_25_H_31_ClFN_8_O_9_P_2_ 703.1362, found 703.1335 [M-H]^-^.

1-[(2*R*,3*R*,4*S*,5*R*)-3,4-Dihydroxy-5-(hydroxymethyl)tetrahydrofuran-2-yl]-4-{[(4-ethynylbenzyl)oxy]imino}-3-methyl-3,4-dihydropyrimidin-2(1*H*)-one (9)

O-(4-Ethynylbenzyl)hydroxylamine hydrochloride (214 mg, 1.28 mmol, 1.0 eq.) and cytidine (**8**, 621 mg, 2.55 mmol, 2.0 eq.) were suspended in pyridine, and the mixture was stirred at 100 °C for 4 d. The solvent was removed *in vacuo,* and the remaining pyridine was co-evaporated with water (50 mL), toluene (50 mL), and ethanol (50 mL). The residue was purified by column chromatography using 5 % H_3_COH in CH_2_Cl_2_ to 10 % H_3_COH in CH_2_Cl_2_ to obtain the product as a pale yellow solid (237 mg, 0.63 mmol, 50 %). *R*_*f*_ (10 % methanol in CH_2_Cl_2_): 0.33. Purity (HPLC-A): 96 % (*R*_*t*_ = 6.9 min). mp (140 °C🡪200 °C, 1 °C/min): decomposition over 163 °C. HRMS (APCI): m/z = calcd. for C_18_H_20_N_3_O_6_ 374.1347, found 374.1329 [M+H]^+^.

^1^H NMR (CD_3_OD, 600 MHz): δ (ppm) = 7.45-7.42 (m, 2H, 3-C*H*_phenyl_ and 5-C*H*_phenyl_), 7.36-7.34 (m, 2H, 2-C*H*_phenyl_ and 6-C*H*_phenyl_), 7.21 (d, *J* = 8.2 Hz, 1H, 6-C*H*_pyrimidine_), 5.85 (d, *J* = 5.5 Hz, 1H, 1ꞌ-C*H*), 5.55 (d, *J* = 8.2 Hz, 1H, 5-C*H*_pyrimidine_), 5.02 (s, 2H, C*H*_2-benzyl_), 4.14 (t, *J* = 5.5 Hz, 1H, 2ꞌ-C*H*), 4.10 (dd, *J* = 5.5, 3.9 Hz, 1H, 3ꞌ-C*H*), 3.94 (h, *J* = 3.3 Hz, 1H, 4ꞌ-C*H*), 3.77 (dd, *J* = 12.1, 2.9 Hz, 1H, 5ꞌ-C*H*H), 3.68 (dd, *J* = 12.1, 3.4 Hz, 1H, 5ꞌ-CH*H*), 3.46 (s, 1H, C*H*_ethynyl_).^13^C NMR (CD_3_OD, 151 MHz): δ (ppm) = 151.5 (*C*=O_pyrimidine_), 146.8 (*C*=N_pyrimidine_), 140.4 (*C*-1_phenyl_), 133.1 (*C*-6_pyrimidine_), 133.0 (2C, *C*-3_phenyl_ and *C*-5_phenyl_), 128.9 (2C, *C*-2_phenyl_ and *C*-6_phenyl_), 123.1 (*C*-4_phenyl_), 98.6 (*C*-5_pyrimidine_), 89.8 (*C*-1ꞌ), 86.2 (*C*-4ꞌ), 84.3 (*C*q_ethynyl_), 78.7 (*C*H_ethynyl_), 76.0 (*C*H_2-benzyl_), 74.8 (*C*-2ꞌ), 71.7 (*C*-3ꞌ), 62.7 (*C*-5ꞌ). FTIR (neat): ṽ (cm^-1^) = 3356 (m, C-H, alkynyl), 3278 (bm, O-H and N-H), 3098 (w, C-H, aromatic), 2924, 2874 (w, C-H, aliphatic), 1682, 1663 (s, C=O and C=N), 1605 (m, C=C, pyrimidine), 1504 (w, C=C, aromatic).

Next, the product (117 mg, 0.31 mmol, 1.0 eq.), iodomethane (39 µL, 89 mg, 0.63 mmol, 2.0 eq.) and K_2_CO_3_ (87 mg, 0.63 mmol, 2.0 eq.) were suspended in DMF and mixture was stirred at 74 °C for 4 d. The mixture was filtrated, the residue was washed with acetone (40 mL), and the filtrate's solvent was removed *in vacuo*. The residue was purified by column chromatography using 7 % H_3_COH in CH_2_Cl_2_ to obtain the product as pale yellow solid (67 mg, 0.17 mmol, 56 %). *R*_*f*_ (10 % methanol in CH_2_Cl_2_): 0.41. Purity (HPLC-A): 97 % (*R*_*t*_ = 9.0 min). mp (170 °C🡪230 °C, 1 °C/min): 183.0 °C. HRMS (APCI): m/z = calcd. for C_19_H_22_N_3_O_6_ 388.1503, found 388.1496 [M+H]^+^. FTIR (neat): ṽ (cm^-1^) = 3364 (C-H, alkynyl), 3287, 3225 (m, O-H), 2967, 2862 (w, C-H, aliphatic), 2106 (w, C≡C), 1662 (s, C=O and C=N), 1577 (m, C=C, pyrimidine), 1508 (w, C=C, aromatic). Purity (HPLC-A): >99 % (*R*_*t*_ = 9.3 min).^1^H NMR (CD_3_OD, 600 MHz): δ (ppm) = 7.44-7.42 (m, 2H, 3-C*H*_phenyl_ and 5-C*H*_phenyl_), 7.35-7.32 (m, 2H, 2-C*H*_phenyl_ and 6-C*H*_phenyl_), 7.30 (d, *J* = 8.4 Hz, 1H, 6-C*H*_pyrimidine_), 6.24 (d, *J* = 8.3 Hz, 1H, 5-C*H*_pyrimidine_), 5.87 (d, *J* = 5.0 Hz, 1H, 1ꞌ-C*H*), 4.99 (s, 2H, C*H*_2-benzyl_), 4.14-4.12 (m, 1H, 2ꞌ-C*H*), 4.10 (dd, *J* = 5.4, 4.1 Hz, 1H, 3ꞌ-C*H*), 3.96-3.93 (m, 1H, 4ꞌ-C*H*), 3.79 (dd, *J* = 12.2, 2.9 Hz, 1H, 5ꞌ-C*H*H), 3.70 (dd, *J* = 12.2, 3.5 Hz, 1H, 5ꞌ-CH*H*), 3.46 (s, 1H, C*H*_ethynyl_), 3.18 (s, 3H, CH_3_). ^1^H NMR (DMSO-d_6_, 600 MHz): δ (ppm) = 7.49-7.43 (m, 2H, 3-C*H*_phenyl_ and 5-C*H*_phenyl_), 7.36 (dd, *J* = 8.4, 2.9 Hz, 3H, 2-C*H*_phenyl_ and 6-C*H*_phenyl_ and 6-C*H*_pyrimidine_), 6.14 (d, *J* = 8.3 Hz, 1H, 5-C*H*_pyrimidine_), 5.78 (d, *J* = 5.7 Hz, 1H, 1ꞌ-C*H*), 5.28 (d, *J* = 5.8 Hz, 1H, 2ꞌ-O*H*), 5.04 (d, *J* = 5.0 Hz, 1H, 3ꞌ-O*H*), 5.01 (t, *J* = 5.2 Hz, 1H, 5ꞌ-O*H*), 4.98 (s, 2H, C*H*_2-benzyl_), 4.16 (s, 1H, C*H*_ethynyl_), 3.97 (q, *J* = 5.6 Hz, 1H, 2ꞌ-C*H*), 3.93 (td, *J* = 5.1, 3.7 Hz, 1H, 3ꞌ-C*H*), 3.80 (q, *J* = 3.5 Hz, 1H, 4ꞌ-C*H*), 3.58 (ddd, *J* = 11.9, 5.3, 3.4 Hz, 1H, 5ꞌ-C*H*H), 3.52 (ddd, *J* = 11.9, 5.1, 3.5 Hz, 1H, 5ꞌ-CH*H*), 3.07 (s, 3H, CH_3_).^13^C NMR (CD_3_OD, 151 MHz): δ (ppm) = 151.9 (*C*=O_pyrimidine_), 151.5 (*C*=N_pyrimidine_), 140.6 (*C*-1_phenyl_), 133.3 (*C*-6_pyrimidine_), 132.9 (2C, *C*-3_phenyl_ and *C*-5_phenyl_), 129.1 (2C, *C*-2_phenyl_ and *C*-6_phenyl_), 123.0 (*C*-4_phenyl_), 93.8 (*C*-5_pyrimidine_), 90.8 (*C*-1ꞌ), 86.1 (*C*-4ꞌ), 84.3 (*C*q_ethynyl_), 78.6 (*C*H_ethynyl_), 76.3 (*C*H_2-benzyl_), 75.1 (*C*-2ꞌ), 71.5 (*C*-3ꞌ), 62.6 (*C*-5ꞌ), 29.3 (CH_3_) ^13^C NMR (DMSO-d_6_, 151 MHz): δ (ppm) = 149.8 (*C*=N_pyrimidine_), 149.6 (*C*=O_pyrimidine_), 139.3 (*C*-1_phenyl_), 132.6 (*C*-6_pyrimidine_), 131.6 (2C, *C*-3_phenyl_ and *C*-5_phenyl_), 128.0 (2C, *C*-2_phenyl_ and *C*-6_phenyl_), 120.8 (*C*-4_phenyl_), 91.9 (*C*-5_pyrimidine_), 88.1 (*C*-1ꞌ), 84.6 (*C*-4ꞌ), 83.4 (*C*q_ethynyl_), 80.8 (*C*H_ethynyl_), 74.4 (*C*H_2-benzyl_), 73.1 (*C*-2ꞌ), 70.0 (*C*-3ꞌ), 61.1 (*C*-5ꞌ), 28.6 (CH_3_).

(2*R*,3*R*,4*R*,5*R*)-2-(Acetoxymethyl)-5-(4-{[(4-ethynylbenzyl)oxy]imino}-3-methyl-2-oxo-3,4-dihydropyrimidin-1(2*H*)-yl)tetrahydrofuran-3,4-diyl diacetate (10)

Compound **9** (481 mg, 1.24 mmol, 1.0 eq.) was dissolved in dry pyridine (10 mL), and acetic acid anhydride (0.41 mL, 444 mg, 4.35 mmol, 3.5 eq.) was added at room temperature. The solution was stirred overnight, methanol (1 mL) was added, and the solution was stirred at room temperature for 1 h. The solvent was removed *in vacuo* and co-evaporated first with toluene (10 mL) and then ethanol (10 mL). The residue was dissolved in CH_2_Cl_2_ (50 mL), and the organic layer was washed with water (2x25 mL) and brine (25 mL). The organic layer was dried over Na_2_SO_4_. Residue was purified by column chromatography using 0 % 🡪 1 % methanol in CH_2_Cl_2_ to obtain the product as pale yellow, sticky solid (602 mg, 1.17 mmol, 95 %). Purity (HPLC-A): 90 % (*R*_*t*_ = 16.4 min). *R*_*f*_ (1 % methanol in CH_2_Cl_2_): 0.45. HRMS (ESI): m/z = calcd. for C_25_H_28_N_3_O_9_ 514.1820, found 514.1818 [M+H]^+^. ^1^H NMR (CD_3_OD, 600 MHz): δ (ppm) = 7.44-7.42 (m, 2H, 3-C*H*_phenyl_ and 5-C*H*_phenyl_), 7.35-7.33 (m, 2H, 2-C*H*_phenyl_ and 6-C*H*_phenyl_), 7.02 (d, *J* = 8.4 Hz, 1H, 6-C*H*_pyrimidine_), 6.29 (d, *J* = 8.3 Hz, 1H, 5-C*H*_pyrimidine_), 5.89 (d, *J* = 5.2 Hz, 1H, 1ꞌ-C*H*), 5.41 (dd, *J* = 6.1, 5.1 Hz, 1H, 2ꞌ-C*H*), 5.37 (dd, *J* = 6.1, 5.0 Hz, 1H, 3ꞌ-C*H*), 5.00 (s, 2H, C*H*_2-benzyl_), 4.32 (dd, *J* = 3.9, 1.7 Hz, 2H, 5ꞌ-C*H*_*2*_), 4.28 (dt, *J* = 5.0, 3.9 Hz, 1H, 4ꞌ-C*H*), 3.46 (s, 1H, C*H*_ethynyl_), 3.17 (s, 3H, C*H*_3 pyrimidine_), 2.09(8) (s, 3H, 3ꞌ-C*H*_3 acetate_), 2.09(5) (s, 3H, 5ꞌ-C*H*_3 acetate_), 2.06 (s, 3H, 2ꞌ-C*H*_3 acetate_). ^13^C NMR (CD_3_OD, 151 MHz): δ (ppm) = 172.2 (*C*=O-5ꞌ_acetate_), 171.4 (2C, *C*=O-2ꞌ_acetate_ and *C*=O-3ꞌ_acetate_), 151.4 (*C*=O_pyrimidine_), 151.0 (*C*=N_pyrimidine_), 140.5 (*C*-1_phenyl_), 133.2 (*C*-6_pyrimidine_), 132.9 (2C, *C*-3_phenyl_ and *C*-5_phenyl_), 129.1 (2C, *C*-2_phenyl_ and *C*-6_phenyl_), 123.0 (*C*-4_phenyl_), 94.5 (*C*-5_pyrimidine_), 90.3 (*C*-1ꞌ), 84.3 (*C*q_ethynyl_), 80.8 (*C*-4ꞌ), 78.6 (*C*H_ethynyl_), 76.4 (*C*H_2-benzyl_), 73.8 (*C*-2ꞌ), 71.7 (*C*-3ꞌ), 64.4 (*C*-5ꞌ), 29.3 (*C*H_3 pyrimidine_), 20.7 and 20.4 (2C, *C*H_3_-3ꞌ_acetate_ and *C*H_3_-5ꞌ_acetate_), 20.3 (*C*H_3_-2ꞌ_acetate_). FTIR (neat): ṽ (cm^-1^) = 3271 (w, C-H, alkynyl), 2932, 2870 (w, C-H, aliphatic), 1744 (s, C=O, acetate), 1686, 1662 (s, C=O and C=N, pyrimidine), 1585 (m, C=C, pyrimidine), 1504 (w, C=C, aromatic).

1-[(2*R*,3*R*,4*S*,5*R*)-3,4-Dihydroxy-5-(hydroxymethyl)tetrahydrofuran-2-yl]-4-[({4-[1-(2-fluoroethyl)-1*H*-1,2,3-triazol-4-yl]benzyl}oxy)imino]-3-methyl-3,4-dihydropyrimidin-2(1*H*)-one (11)

2-Fluroethanol (191 µL, 210 mg, 3.28 mmol, 3.72 eq.), 4-toluenesulfonyl chloride (687 mg, 3.61 mmol, 4.10 eq.) and KOH (276 mg, 4.92 mmol, 5.59 eq.) were suspended in dry CH_2_Cl_2_ (5 mL), and the mixture was stirred at room temperature for 3 h. CH_2_Cl_2_ (22 mL) was added, and the mixture was extracted with water (2x25 mL). The organic layer was dried over Na_2_SO_4,_ and the solvent was removed, yielding crude 2-fluoroethyl 4-methylbenzenesulfonate as a pale yellow liquid (734 mg, 3.36 mmol, 103 %), which was used without further purification

^1^H NMR (CDCl_3_, 400 MHz, 367M): δ (ppm) = 7.85-7.76 (m, 2-C*H*_tosyl_ and 6-C*H*_tosyl_), 7.40-7.31 (m, 2H, 3-C*H*_tosyl_ and 5-C*H*_tosyl_), 4.66-4.61 (m, 1H, FC*H*HCH_2_O), 4.53-4.49 (m, 1H, FCH*H*CH_2_O), 4.33-4.28 (m, 1H, FCH_2_C*H*HO), 4.25-4.21 (m, 1H, FCH_2_CH*H*O), 2.46 (s, *3*H, C*H*_3_). ^13^C NMR (CDCl_3_, 101 MHz, 367M): δ (ppm) = 145.3 (*C*-4_tosyl_), 132.8 (*C*-1_tosyl_), 130.1 (2C, *C*-3_tosyl_ and *C*-5_tosyl_), 128.1 (2C, *C*-2_tosyl_ and *C*-6_tosyl_), 80.7 (d, *J* = 173.6 Hz, F*C*H_2_CH_2_O), 68.6 (d, *J* = 20.9 Hz, FCH_2_*C*H_2_O), 21.8 (CH_3_). ^19^F NMR (CDCl_3_, 376 MHz, 367M): δ (ppm) = -224.7 (tt, *J* = 47.1, 27.1 Hz).

2-Fluoroethyl 4-methylbenzenesulfonate was dissolved in dry DMF (50 mL), and NaN_3_ (1.28 g, 19.67 mmol, 22.35 eq.) was added. The mixture was stirred at 60 °C for 2 d and was then filtrated. CuI (624 mg, 3.28 mmol, 4.10 eq.), sodium ascorbate (650 mg, 3.28 mmol, 4.10 eq.) and triethylamine (0.45 mL, 332 mg, 3.28 mmol, 4.10 eq.), followed by compound **10** (451 mg, 0.88 mmol, 1.0 eq.) in DMF (5 mL) were added to the filtrate. The mixture was stirred at room temperature overnight, controlled by LC/MS. The solvent was removed in vacuo, and the residue was suspended in ethyl acetate (100 mL). The organic layer was washed with aqueous sodium tartrate (1 M, 2x50 mL) and water (50 mL). Organic layer was dried over Na_2_SO_4_, and the solvent was removed *in vacuo*, yielding crude (2*R*,3*R*,4*R*,5*R*)-2-(acetoxymethyl)-5-{4-[({4-[1-(2-fluoroethyl)-1*H*-1,2,3-triazol-4-yl]benzyl}oxy)imino]-3-methyl-2-oxo-3,4-dihydropyrimidin-1(2*H*)-yl}tetrahydrofuran-3,4-diyl diacetate (516 mg, 0.86 mmol, 97 %), which was used without further purification. The intermediate was dissolved in methanol, and NH_3_ (4 M in methanol, 3.3 mL, 13.2 mmol, 15 eq.) was added. The mixture was stirred overnight, controlled by LC/MS. The solvent was removed *in vacuo*, and the residue was purified *via* semi-preparative C18-HPLC (RP-HPLC-C), yielding the product (132 mg, 0.34 mmol, 39 %) as a pale yellow solid. *R*_*t*_ (RP-HPLC-C): 30.5-32.5 min: solvent A: CH_3_CN, solvent B: water, 0-2 min: 15 % solvent A, 3 mL/min to 5 mL/min, 2-50 min: 15 % to 100 % solvent A, 50-55 min: 100 % solvent A, 55-56 min: 100 % to 15 % solvent A, 56-60 min: 15 % solvent A, 60-61 min: 15 % solvent A, 5 mL/min to 0.1 mL/min, Injection volume: 800 µL. Purity (HPLC-A, 254 nm): 96 % (*R*_*t*_ = 7.4 min). HRMS (ESI): m/z = calcd. for C_21_H_26_FN_6_O_6_ 477.1892, found 477.1930 [M+H]^+^. ^1^H NMR (CD_3_OD, 400 MHz): δ (ppm) = 8.35 (s, 1H, C*H*_triazole_), 7.98 (s, 2H, C*H*_DMF_), 7.83-7.79 (m, 2H, 3-C*H*_phenyl_ and 5-C*H*_phenyl_), 7.47-7.43 (m, 2H, 2-C*H*_phenyl_ and 6-C*H*_phenyl_), 7.29 (d, *J* = 8.4 Hz, 1H, 6-C*H*_pyrimidine_), 6.26 (d, *J* = 8.3 Hz, 1H, 5-C*H*_pyrimidine_), 5.87 (d, *J* = 4.9 Hz, 1H, 1ꞌ-C*H*), 5.02 (s, 2H, C*H*_2-benzyl_), 4.92 (dd, *J* = 5.3, 4.1 Hz, 1H, CH_2_C*H*HF), 4.82-4.78 (m, 2H, CH_2_CH*H*F and C*H*HCH_2_F), 4.76-4.73 (m, 1H, CH*H*CH_2_F), 4.15-4.09 (m, 2H, 2ꞌ-C*H* and 3ꞌ-C*H*), 3.94 (q, *J* = 3.4 Hz, 1H, 4ꞌ-C*H*), 3.79 (dd, *J* = 12.2, 2.9 Hz, 1H, 4ꞌ-C*H*H), 3.69 (dd, *J* = 12.1, 3.5 Hz, 1H, 4ꞌ-CH*H*), 3.19 (s, 3H, C*H*_3 pyrimidine_), 2.99 (s, 6H, C*H*_3 DMF_), 2.86 (d, *J* = 0.7 Hz, 3H, C*H*_3 DMF_). ^13^C NMR (CD_3_OD, 101 MHz): δ (ppm) = 164.9 (*C*=O_DMF_), 151.9 (*C*=O_pyrimidine_), 151.5 (*C*=N_pyrimidine_), 148.9 (*C*-1_triazole_), 140.0 (*C*-1_phenyl_), 133.2 (*C*-6_pyrimidine_), 131.0 (*C*-4_phenyl_), 129.9 (2C, *C*-3_phenyl_ and *C*-5_phenyl_), 126.6 (2C, *C*-2_phenyl_ and *C*-6_phenyl_), 122.9 (*C*-2_triazole_), 93.9 (*C*-5_pyrimidine_), 90.8 (*C*-1ꞌ), 86.1 (*C*-4ꞌ), 82.8 (d, *J* = 170.8 Hz, *C*H_2_F), 76.5 (*C*H_2-benzyl_), 75.0 (*C*-2ꞌ), 71.5 (*C*-3ꞌ), 62.7 (*C*-5ꞌ), 52.0 (d, *J* = 20.3 Hz, *C*H_2_CH_2_F), 37.0 (2C, *C*H_3 DMF_), 31.7 (2C, *C*H_3 DMF_), 29.3 (CH_3 pyrimidine_). ^19^F NMR (CD_3_OD, 376 MHz): δ (ppm) = -224.5 (tt, *J* = 46.8, 27.0 Hz). FTIR (neat): ṽ (cm^-1^) = 3345 (bm, N-H and O-H), 3140 (w, C-H, alkene), 2963 and 2924 (w, C-H, aliphatic), 1659 (s, C=O and C=N), 1585 (m, C=C, pyrimidine), 1497 (w, C=C, aromatic).

{[{[(2*R*,3*S*,4*R*,5*R*)-5-(-4-{[(4-Ethynylbenzyl)oxy]imino}-3-methyl-2-oxo-3,4-dihydropyrimidin-1(2*H*)-yl)-3,4-dihydroxytetrahydrofuran-2-yl]methoxy}(hydroxy)phosphoryl]methyl}phosphonic acid (12)

1-[(2*R*,3*R*,4*S*,5*R*)-3,4-Dihydroxy-5-(hydroxymethyl)tetrahydrofuran-2-yl]-4-{[(4-ethynylben-zyl)oxy]imino}-3-methyl-3,4-dihydropyrimidin-2(1*H*)-one (**9**, 208 mg, 0.38 mmol, 1.0 eq.) was suspended in trimethyl phosphate (2 mL, 0 °C), and methylenebis(phosphonic dichloride) (143 mg, 0.57 mmol, 1.5 eq.) in trimethyl phosphate (2 mL, 0 °C) was added. The mixture was stirred at 0 °C (controlled by LC-MS) for 85 min, and triethylammonium acetate buffer (TEAA, 0.1 M in water, pH = 7, 40 mL) was added. The solution was neutralized by the addition of triethylamine and was then lyophilized. The compound was then pre-purified by RP-HPLC-A and purified by RP-HPLC-B. Suitable fractions were pooled and lyophilized to obtain the final product as a glassy colorless solid (1.17 eq. Et_3_N-salt [663.44 g/mol], 46 mg, 69 µmol, 13 %). *R*_*t*_ (RP-HPLC-A): 27.7-29.5 min: solvent A: CH_3_CN, solvent B: 0.1 M TEAA, 0-2 min: 25 % solvent A, 3 mL/min 🡪 5 mL/min, 2-30 min: 40 % solvent A, 30-40 min: 40 % solvent A, 40-41 min: 40 % 🡪 100 % solvent A, 41-51 min: 100 % solvent A, 51-52 min: 100 % to 25 % solvent A, 52-59 min: 25 % solvent A, 59-60 min: 25 % solvent A, 5 mL/min to 0.1 mL/min, Injection volume: 300 µL. *R*_*t*_ (RP-HPLC-B): 31.0-33.0 min: solvent A: CH_3_CN, solvent B: 0.1 M TEAA, 0-2 min: 25 % solvent A, 3 mL/min to 5 mL/min, 2-50 min: 40 % solvent A, 50-52 min: 40 % to 25 % solvent A, 52-59 min: 25 % solvent A, 59-60 min: 25 % solvent A, 5 mL/min to 0.1 mL/min, Injection volume : 400-600 µL. Purity determined by HPLC (210 nm) >99 % (*R*_*t*_ = 12.2 min). Exact mass (ESI): m/z = calcd. for C_20_H_24_N_3_O_11_P_2_ 544.0892, found 544.0862 [M-H]^-^.^1^H NMR (D_2_O, 400 MHz): δ (ppm) = 7.48 (d, *J* = 8.2 Hz, 2H, 3-C*H*_phenyl_ and 5-C*H*_phenyl_), 7.37 (d, *J* = 8.2 Hz, 2H, 2-C*H*_phenyl_ and 6-C*H*_phenyl_), 7.32 (d, *J* = 8.3 Hz, 1H, 6-C*H*_pyrimidine_), 6.38 (d, *J* = 8.3 Hz, 1H, 5-C*H*_pyrimidine_), 5.89 (d, *J* = 4.8 Hz, 1H, 1ꞌ-C*H*), 4.97 (s, 2H, C*H*_2-benzyl_), 4.34-4.25 (m, 2H, 2ꞌ-C*H* and 3ꞌ-C*H*), 4.22-4.19 (m, 1H, 4ꞌ-C*H*), 4.18-4.09 (m, 2H, 5ꞌ-C*H*_2_), 3.52 (s, 1H, C*H*_ethynyl_), 3.18 (q, *J* = 7.3 Hz, 7H, C*H*_2_ _NEt3_), 3.11 (s, *3*H, C*H*_3 pyrimidine_), 2.32 (t, *J* = 19.4 Hz, 2H, PC*H*_2_P), 1.26 (t, *J* = 7.3 Hz, 10.5H, C*H*_2_ _NEt3_). ^13^C NMR (D_2_O, 101 MHz): δ (ppm) = 153.6 (*C*=N_pyrimidine_), 151.0 (*C*=O_pyrimidine_), 138.0 (*C*-1_phenyl_), 133.3 (*C*-6_pyrimidine_), 132.26 (2C, *C*-3_phenyl_ and *C*-5_phenyl_), 128.8 (2C, *C*-2_phenyl_ and *C*-6_phenyl_), 121.3 (*C*-4_phenyl_), 93.8 (*C*-5_pyrimidine_), 88.8 (*C*-1ꞌ), 83.7 (*C*q_ethynyl_), 83.0 (d, *J* = 7.0 Hz, *C*-4ꞌ), 78.7 (*C*H_ethynyl_), 75.2 (*C*H_2-benzyl_), 73.2 (*C*-2ꞌ), 69.8 (*C*-3ꞌ), 63.5 (bs, *C*-5ꞌ), 46.7 (3.5C, *C*H_2 NEt3_), 29.3 (CH_3_), 26.7 (t, *J* = 127.5 Hz, P*C*H_2_P), 8.3 (3.5C, *C*H_3 NEt3_). ^31^P NMR (D_2_O/D_3_PO_4_, 162 MHz): δ (ppm) = 17.8 (bs, 2P). 

[((2*R*,3*S*,4*R*,5*R*)-5-{4-[({4-[1-(2-Fluoroethyl)-1*H*-1,2,3-triazol-4-yl]benzyl}oxy)-imino]-3-methyl-2-oxo-3,4-dihydropyrimidin-1(2*H*)-yl}-3,4-dihydroxytetrahydro-furan-2-yl)methoxy]-(phosphonomethyl)phosphonic acid (MRS-4648 (2))

Compound **11** (51 mg, 0.13 mmol, 1.0 eq.) was dissolved in trimethyl phosphate (3 mL), and the solution was cooled to 0 °C. Methylenebis(phosphonic dichloride) (50 mg, 0.20 mmol, 1.5 eq.) was added, and the mixture was stirred at 0 °C for 50 min (controlled by LC-MS). Triethylammonium acetate (1 M in water, pH = 7, 1 mL) was added, and the solution was stirred at 0 °C for 30 min and for 1 h at room temperature. The crude product was then purified *via* semi-preparative RP-HPLC (RP-HPLC-J). Fractions containing the product were pooled and lyophilized to obtain the final product as a colorless solid (41 mg, 0.041 mmol, 32 % [M = 998.08 g/mol, 1.0 eq. acetic acid and 3.0 eq. Et_3_N-salt]). *R*_*t*_ (RP-HPLC-J): 37.5-39 min: solvent A: CH_3_CN, solvent B: 0.1 M TEAA, 0-2 min: 15 % solvent A, 3 mL/min 🡪 5 mL/min, 2-30 min: 15 % 🡪 30 % solvent A, 30-35 min: 30 % 🡪 100 % solvent A, 35-40 min: 100 % solvent A, 40-45 min: 100 % 🡪 15 % solvent A, 45-55 min: 15 % solvent A, 55-57 min: 15 % solvent A, 5 mL/min 🡪 0.1 mL/min, Injection volume: 800 µL. column: Agilent technologies, PolarisTM C18-A, 250x21.2 mm, 5 µm, precolumn: Agilent technologies, PursuitTM C18, 30x21.2 mm, 5 µm. Purity (HPLC-A): 99 % (*R*_*t*_ = 11.3 min). HRMS (ESI): m/z = calcd. for C_22_H_28_FN_6_O_11_P_2_ 633.1273, found 633.1261 [M-H]^-^. ^1^H NMR (D_2_O, 400 MHz): δ (ppm) = 8.38-8.35 (m, 1H, C*H*_triazole_), 7.79 (dd, *J* = 8.2, 2.2 Hz, 2H, 3-C*H*_phenyl_, 5-C*H*_phenyl_), 7.58-7.50 (m, 2H, 2-C*H*_phenyl_, 6-C*H*_phenyl_), 7.32 (dd, *J* = 8.5, 1.1 Hz, 1H, 6-C*H*_pyrimidine_), 6.44 (dd, *J* = 8.4, 1.5 Hz, 1H, 5-C*H*_pyrimidine_), 5.92 (d, *J* = 5.0 Hz, 1H, 1ꞌ-C*H*), 5.03 (s, 2H, C*H*_2-benzyl_), 4.95 (d, *J* = 4.6 Hz, 1H, CH_2_C*H*HF), 4.87-4.81 (m, 2H, CH_2_CH*H*F, C*H*HCH_2_F), 4.77 (m, 1H, CH*H*CH_2_F [below water signal]), 4.35-4.28 (m, 2H, 2ꞌ-C*H*, 3ꞌ-C*H*), 4.22-4.17 (m, 1H, 4ꞌ-C*H*), 4.10 (dd, *J* = 5.2, 2.9 Hz, 2H, 4ꞌ-C*H*_2_), 3.18 (q, *J* = 7.5 Hz, 21H, C*H*_3 pyrimidine_, C*H*_3 NEt3_), 2.17 (td, *J* = 19.8, 1.4 Hz, 2H, PC*H*_2_P), 1.92 (s, 3H, C*H*_3 acetyl_), 1.26 (t, *J* = 7.4 Hz, 27H, C*H*_2 NEt3_). ^13^C NMR (D_2_O, 101 MHz): δ (ppm) = 181.2 (1C, *C*=O_acetyl_), 153.8 (1C, *C*=O_pyrimidine_), 151.2 (1C, *C*=N_pyrimidine_), 147.4 (1C, *C*-1_triazole_), 137.6 (1C, *C*-1_phenyl_), 132.7 (1C, *C*-6_pyrimidine_), 129.6 (2C, *C*-3_phenyl_, *C*-5_phenyl_), 129.4 (1C, *C*-4_phenyl_), 125.8 (2C, *C*-2_phenyl_, *C*-6_phenyl_), 122.9 (1C, *C*-2_triazole_), 94.1 (1C, *C*-5_pyrimidine_), 88.5 (1C, *C*-1ꞌ), 83.2 (d, *J* = 7.9 Hz, 1C, *C*-4ꞌ), 82.2 (d, *J* = 167.0 Hz, 1C, *C*H_2_F), 75.2 (1C, *C*H_2-benzyl_), 73.1 (1C, *C*-2ꞌ), 70.0 (1C, *C*-3ꞌ), 63.7 (1C, *C*-5ꞌ), 50.9 (d, *J* = 19.4 Hz, 1C, *C*H_2_CH_2_F), 46.8 (9C, 1C, *C*H_2 NEt3_), 29.3 (1C, *C*H_3 pyrimidine_), 27.5 (m, 1C, P*C*H_2_P), 23.2 (1C, *C*H_3 acetyl_), 8.3 (9C, *C*H_3 NEt3_). ^19^F NMR (D_2_O, 376 MHz): δ (ppm) = -222.3 (tt, *J* = 46.4, 28.2 Hz, 1F). ^31^P NMR (D_2_O/D_3_PO_4_): δ (ppm) = 18.3 (d, *J* = 9.7 Hz, 1P, α-*P*), 14.7 (d, *J* = 9.8 Hz, 1P, β-*P*). 

Radiochemistry. General Methods. 

The first step of the radiosynthesis was carried out on a modified PET tracer radio synthesizer (TRACERLab Fx_FDG_, GE Healthcare). The recorded data was processed by the TRACERLab Fx software (GE Healthcare). Separation and purification of the radiolabeled compounds were performed on the semipreparative radio-HPLC system A: K-500 and K-501 pump, K-2000 UV detector (Herbert Knauer GmbH), NaI(TI) Scintibloc 51 SP51 γ-detector (Crismatec) and an ACE 5 AQ column (250 mm × 10 mm). Method A started with a linear gradient from 10% to 90% CH_3_CN in water (0.1% TFA) over 30 min, holding for 5 min and followed by a linear gradient from 90% to 10% CH_3_CN in water (0.1% TFA) over 5 min, with l = 254 nm and a flow rate of 5.0 mL min^-1^. Radiochemical purities and molar activities were determined using the analytical radio-HPLC system B: Two Smartline 1000 pumps and a Smartline UV detector 2500 (Herbert Knauer GmbH), a GabiStar γ-detector (Raytest Isotopenmessgeräte GmbH) and a Nucleosil 100-5 C-18 column (250 mm × 4 mm). Method B started with a linear gradient from 10% to 100% CH_3_CN in water (0.1% TFA) over 15 min, holding for 3 min followed by a linear gradient from 100% to 10% CH_3_CN in water (0.1% TFA) over 2 min, with l = 254 nm and a flow rate of 1.0 mL min^-1^. The recorded data of both HPLC-systems were processed by the GINA Star software (Raytest Isotopenmessgeräte GmbH). No-carrier-added aqueous [^18^F]fluoride was produced on a RDS 111e cyclotron (CTI-Siemens) by irradiation of a water target (2.8 mL) using 10 MeV proton beams on 97.0% enriched [^18^O]H_2_O by the ^18^O(p,n)^18^F nuclear reaction.

[^18^F]-[((2*R*,3*S*,4*R*,5*R*)-5-{2-chloro-6-[(4-{1-[2-(fluoro)ethyl]-1*H*-1,2,3-triazol-4-yl}benzyl)-(propyl)amino]-9*H*-purin-9-yl}-3,4-dihydroxytetrahydrofuran-2-yl)methoxy]methylene-bisphosphonic acid ([^18^F]PSB-19427, [^18^F]1)

In a computer controlled TRACERLab Fx_FDG_ Synthesizer a batch of aqueous [^18^F]fluoride (3.1 – 5.2 GBq) from the cyclotron target was passed through an anion exchange resin (pre-conditioned Sep-Pak® Light QMA cartridge with carbonate counter-ion). [^18^F]fluoride was eluted from the resin with a mixture of 1 M K_2_CO_3_ (aqueous, 40 µL), water for injection (WFI, 200 µL), and acetonitrile (800 µL, DNA-grade) containing Kryptofix®2.2.2 (K_2.2.2_, 20 mg, 53 µmol) in the reactor. Subsequently, the aqueous K(K_2.2.2_)[^18^F]fluoride solution was carefully evaporated to dryness *in vacuo*. An amount of precursor compound 2-azidoethyl 4-methylbenzenesulfonate (20 mg, 83 µmol) in acetonitrile (DNA-grade, 500 µL) was added and the mixture was heated at 110°C for 3 min. Meanwhile, the labeled 1-azido-2-[^18^F]fluoroethane was distilled from the reactor into an ice-cooled 10 mL flask that contained a mixture of **3** (5.0 mg, 8.1 µmol) in DMF (300 µL), CuSO_4_·5H_2_O (40 mg, 160 µmol) in HEPES buffer (pH: 5.7, 100 µL) and sodium ascorbate (63 mg, 318 µmol) in HEPES buffer (pH: 5.7, 100 µL). After 30 min stirring at 60°C, the mixture was passed through a PTFE sterile filter (0.2 µm). The filter was rinsed with DMF (500 µL) and then with WFI (500 µL). The combined filtrate and rinsing solutions were purified by gradient-radio-HPLC system A (method A). The product fraction of compound [^18^F]PSB-19427 (retention time t_*R*_([^18^F]PSB-19427)= 12.2 min) was collected in a flask pretreated with Sigmacote® and solution was evaporated to dryness *in vacuo*. The residue was redissolved in WFI/EtOH (1 mL, 9:1 v/v). Product compound [^18^F]PSB-19427 was obtained in an overall radiochemical yield of 21.7 ± 3.5% (decay-corrected, based on cyclotron-derived [^18^F]F-ions, n = 21) in 119 ± 10 min from the end of radionuclide production. [^18^F]PSB-19427 was isolated in radiochemical purities of >99% with molar activities in the range of 2.3 – 54.3 GBq/µmol at the end of the synthesis. Radiochemical purities and molar activities of [^18^F]PSB-19427 (retention time t_*R*_([^18^F]PSB-19427)= 9.4 min) were determined by analytical radio-HPLC B (method B). *R*_*t*_ (Radio HPLC-A): 12.2 min. Rcy: 21.7 ± 3.5 %. Rcp (Radio HPLC-B): >99 % (*R*_*t*_ = 9.4 min). Molar activity: 2.3 – 54.3 GBq/µmol. Log *D*_7.4_: -0.12 ± 0.03. PPB: >99 %. Mouse serum: stable over 90 min. Human serum: stable over 90 min.

[^18^F]-{[(2*R*,3*S*,4*R*,5*R*)-5-(4-{[(4-{1-[2-(fluoro)ethyl]-1*H*-1,2,3-triazol-4-yl}benzyl)-oxy]imino}-3-methyl-2-oxo-3,4-dihydropyrimidin-1(2*H*)-yl)-3,4-dihydroxytetra-hydrofuran-2-yl]methoxy}methylenebisphosphonic acid ([^18^F]MRS-4648, [^18^F]2)

In a computer-controlled TRACERLab Fx_FDG_ Synthesizer a batch of aqueous [^18^F]fluoride (3.8 – 5.4 GBq) from the cyclotron target was passed through an anion exchange resin (pre-conditioned Sep-Pak® Light QMA cartridge with carbonate counter-ion). [^18^F]fluoride was eluted from the resin with a mixture of 1 M K_2_CO_3_ (aqueous, 40 µL), water for injection (WFI, 200 µL), and acetonitrile (800 µL, DNA-grade) containing Kryptofix®2.2.2 (K_2.2.2_, 20 mg, 53 µmol) in the reactor. Subsequently, the aqueous K(K_2.2.2_)[^18^F]fluoride solution was carefully evaporated to dryness *in vacuo*. An amount of precursor compound 2-azidoethyl 4-methylbenzenesulfonate (20 mg, 83 µmol) in acetonitrile (DNA-grade, 500 µL) was added and the mixture was heated at 110°C for 3 min. Meanwhile, the labeled 1-azido-2-[^18^F]fluoroethane was distilled from the reactor into an ice-cooled 10 mL flask that contained a mixture of **4** (5.0 mg, 8.1 µmol) in DMF (300 µL), CuSO_4_·5H_2_O (40 mg, 160 µmol) in HEPES buffer (pH: 5.7, 100 µL) and sodium ascorbate (63 mg, 318 µmol) in HEPES buffer (pH: 5.7, 100 µL). After 30 min stirring at 60°C, the mixture was passed through a PTFE sterile filter (0.2 µm). The filter was rinsed with DMF (500 µL) and then with WFI (500 µL). The combined filtrate and rinsing solutions were purified by gradient-radio-HPLC system A (method A). The product fraction of compound [^18^F]MRS-4648 (retention time t_*R*_([^18^F]MRS-4648) = 8.0 min) was collected in a flask pretreated with Sigmacote® and solution was evaporated to dryness *in vacuo*. The residue was redissolved in WFI/EtOH (1 mL, 9:1 v/v). Product compound [^18^F]MRS-4648 was obtained in an overall radiochemical yield of 12.9 ± 3.0% (decay-corrected, based on cyclotron-derived [^18^F]F-ions, n = 8) in 115 ± 15 min from the end of radionuclide production. [^18^F]MRS-4648 was isolated in radiochemical purities of 98 ±1.9% with molar activities in the range of 0.4 – 6.3 GBq/µmol at the end of the synthesis. Radiochemical purities and molar activities of [^18^F]MRS-4648 (retention time t_*R*_([^18^F]MRS-4648)= 8.5 min) were determined by analytical radio-HPLC B (method B). *R*_*t*_ (Radio HPLC-A): 7.9 min. Rcy: 12.9 ± 3.0 %. Rcp (Radio HPLC-B): 98 ±1.9 % (*R*_*t*_ = 8.5 min). Molar activity: 0.4 – 6.3 GBq/µmol. Log *D*_7.4_: 0.74 ± 0.29. PPB: 66.5 %. Mouse serum: stable over 90 min. Human serum: stable over 90 min.

Crystal structure analysis

For crystallization of CD73 in complex with PSB-19427 or JMS04-14 our construct 8.01His of human CD73 was used.^[23]^ This construct is expressed from a derivative of the pHLsec vector, in which the signal peptide of this vector was replaced with the native CD73 signal peptide. The protein was expressed in HEK293S cells in adherent culture in roller bottles. The protein was purified by two chromatography steps. First, the supernatant of the cells was concentrated to 100 mL by ultrafiltration. During ultrafiltration, the culture medium was replaced by a buffer consisting of 50 mM Tris pH 8.0 and 400 mM NaCl. Before application to a 1 mL HisTrap HP column, 4 mL elution buffer (50 mM Tris pH 8.0, 400 mM NaCl, 500 mM imidazole) was added to the 100 mL ultrafiltration concentrate. The HisTrap column was equilibrated with the washing buffer (50 mM Tris pH 8.0, 400 mM NaCl, 20 mM imidazole) before applying the protein solution. After washing the bound protein with 40 mL washing buffer, the protein was eluted with a gradient of 50 mL from washing buffer to the elution buffer. 10 μL 100 mM was added to each fraction tube (fraction sample volume 1 mL) before starting the elution. The pooled fractions were applied to a Superdex 200 16/60 gel filtration column and eluted with the gel filtration buffer consisting of 50 M Tris pH 8.0 and 100 mM NaCl. For crystallization, vapor diffusion trials were set up at 19 °C. 1 µL of the protein solution containing 1 mM of PSB-19427 and 100 µM of ZnCl_2_ or 10 mM of JMS04-14 and 10 μM of ZnCl_2_ were mixed with an equal amount of reservoir buffer solution to give concentrations listed in Table S1. Crystals were stepwise transferred to a cryo buffer composed as reported in Table S1 and finally flash frozen in liquid nitrogen. 

X-ray data collection was carried out at 100 K on beamline 14.1 of the Berlin Synchrotron (BESSY, Berlin, Germany) for CD73×PSB19427 and at EMBL beamline P14 at DESY (Hamburg, Germany) for CD73×JMS04-14. The diffraction data were integrated with XDS^[55]^ via the XDSAPP^[56] ^GUI. The data set of CD73×PSB19427 was scaled with StarAniso due to the strong anisotropic diffraction of the crystals. Coordinates of human CD73 in crystal form IV (pdb code: 6ye1)^[28]^ were taken as the starting model for automatic refinement with buster and pipedream. COOT^[57]^ was used for model building. The two domains of residues 26-333 and 334-551 were used as TLS groups. Structures were validated by MOLPROBITY.^[58]^ Coordinate files and restraint dictionaries for inhibitors were generated using the GRADE web server (http://grade.globalphasing.org/). Polder omit maps^[59]^ were calculated with PHENIX.^[60]^ Relevant crystallographic parameters of the structures are listed in Table S1. Figures were prepared using PyMOL (http://pymol.org).

Determination of the logD_7.4_–values of radiotracers

Triazole radiotracers (~400 kBq) in PBS buffer (10 µL, pH 7.4) were added to PBS buffer (590 µL, pH 7.4) and octan-1-ol (600 µL). The two-layer mixture was shaken for 3 min on a vortex mixer at r.t. and centrifuged (200 g) for 5 min. The main part of the octanol layer (400 µL) was carefully collected and transferred to a new tube containing PBS buffer (400 µL, pH 7.4). The two-layer mixture was shaken again for 10 min and centrifuged for 5 min (200 g). Three samples were prepared, and two aliquots (á 100 µL) of both layers were measured in a gamma-counter 2480 Wizard2 (Perkin-Elmer, Waltham, USA). The partition coefficient was determined by dividing cpm (octanol) by cpm (PBS) and indicated as logD_7.4 _(exp.).

Stability in mouse liver microsomes (MLM)

An aqueous buffer of 75 mM PBS, 12.5 mM MgCl_2_, 0.6 mM NADPH, 1 mg/mL mouse liver microsomes and 5 µM of the respective compound was prepared. 200 µL of these samples were incubated for 90 min at 37 °C and 100 g. Afterward, acetonitrile/methanol (400 µL, 1:1) was added, and the samples were cooled to 0 °C for 10 min. 600 µL buffer was added and the solution was centrifuged for 15 min at 17,880 g. The supernatant was centrifuged again for 15 min at 17,880 g and 4 °C. The obtained solution was diluted with H_2_O/CH_3_CN/CH_3_OH (1:1:1) to a suitable concentration for quantification. Shortly before injection into HPLC, the solution was centrifuged again for 2 min at 17,880 g and 4 °C. The amount of remaining compound in the solution was quantified by ion count in SIM mode via HPLC-MS (CH_3_CN/water). The injection volume was 50 µL. Every experiment was performed in triplicate.

Human serum albumin (HSA) binding. 

Plasma protein binding was determined following previously published procedure.^[38]^

*In vitro* stability in mouse and human serum 

The serum stability of the radioligands [^18^F]MRS-4648 or [^18^F]PSB-19427 was evaluated by incubation in mouse serum at 37°C for up to 90 min. An aliquot of formulated [^18^F]MRS-4648 or [^18^F]PSB-19427 solution (20 μL) was added to a sample of mouse serum (200 μL), and the mixture was incubated at 37°C. Samples of 20 μL each were drawn after periods of 10, 30, 60 and 90 min and quenched in ice-cold acetonitrile (100 µL, DNA-grade) followed by centrifugation (1,100 g) for ≥5 min. The supernatant was analyzed by analytical radio-HPLC B (method B). Serum stability investigations in human serum were performed analogously. HPLC traces are displayed in Fig. S1).

Enzyme inhibition assay 

The CD73 enzyme inhibition assay of recombinant soluble CD73, membrane-bound human CD73, soluble rat CD73 and mouse CD73 was performed as previously described.^[16,35]^

Tissue collection and preparation 

Tumors were obtained from two treatment-naïve breast cancer patients undergoing mastectomy surgery with axillary lymph node dissection at the Department of Plastic and General Surgery at Turku University Hospital (Turku, Finland). The first patient (patient "**X**") was 54-year-old female with infiltrating ductal carcinoma (grade II, hormone receptor positive (ER 95%, PR 30%), Her2 negative, Ki-67 15%, lymph node status 6/55). The second patient (patient "**Y**") was 44-year-old female with infiltrating ductal carcinoma with micropapillary differentiation (grade III, hormone receptor-negative, Her2 positive, Ki-67 25%, lymph node status 4/13). The collection of the tissues is part of an ongoing sample collection performed under the license ETMK 132/2016 with written consent from the patients. The excised primary tumors and metastatic lymph nodes were embedded in the cryo-mold with Tissue-Tek O.C.T. compound (Sakura Finetek Europe BV, The Netherlands), cut at 6 μm onto superfrost glass slides using a cryostat, and stored at -80 °C. 

Immunofluorescence staining

Tumor cryosections were processed for the immunofluorescence analysis of CD73 expression, as described elsewhere.^[41]^ Briefly, the slides were incubated overnight at +4 °C in Shandon Sequenza Staining System (Thermo Scientific) with rabbit anti-human CD73 antibody (h5NT-1_L_, http://ectonucleotidases-ab.com/) diluted at 1:400 in 200 μl PBS containing 2% bovine serum albumin (BSA) and 0.1% (vol/vol) Triton X-100 (blocking buffer). The samples were washed and subsequently incubated for two hours at RT with Alexa Fluor®-633-conjugated goat anti-rabbit antibody (ThermoFisher Life Technologies), diluted in blocking buffer at 1:500. Alexa Fluor® 488-conjugated pan-cytokeratin (catalogue # MA5-18156, ThermoFisher) and Cy3-conjugated anti-smooth muscle cell-α (SMA-α, clone 1A4, Sigma-Aldrich) monoclonal antibodies were added during the incubation with secondary antibody for labeling the epithelial tumor cells and cancer-associated fibroblasts, respectively. The slides were mounted with ProLong® Gold Antifade reagent with DAPI (ThermoFisher) and examined using Zeiss LSM880 confocal microscope with Plan-Apochromat 10×/0.45 objective (Carl Zeiss GmbH, Jena, Germany).

*In situ* enzyme histochemistry 

For the localization of AMPase activities in human tumors, the lead nitrate-based enzyme histochemistry was employed. In brief, tissue cryosections were pre-incubated for 60 min at room temperature in 40 mM Trizma-maleate buffer (TMB, pH 7.3) supplemented with 250 mmol/L sucrose, the alkaline phosphatase inhibitor levamisole (2 mM) and different concentrations of CD73 inhibitors. The enzymatic reaction was then performed for 45 min at 37°C in a final volume of 20 mL of TMB containing 250 mM sucrose, 2 mM levamisole, 1.5 mM Pb(NO_3_)_2_, 1 mM CaCl_2_, 400 μM AMP, and tested CD73 inhibitors at the same concentrations. The lead orthophosphate precipitated in the course of nucleotidase activity was visualized as a brown deposit by incubating sections in 0.5% (NH4)_2_S for 10 s, followed by three washes in TMB for 5 min each. Slides were mounted with Aquatex medium (Merck, Germany). Tissue sections were also stained with hematoxylin and eosin (H&E). Whole slide imaging was performed using Pannoramic P250 Flash slide scanner (3DHistech Ltd., Budapest, Hungary) with a 20× objective. AMPase activity was determined by measuring AMP-specific brown staining intensities from the images using QuPath v.0.3.0 software. Simple tissue detection plugin was used to select whole tissue areas. Default values for hamatoxylin and DAB were used in colour deconvolution (color separation), and DAB channel was used as a proxy for AMPase activity. The average DAB intensity levels were measured as described earlier.^[39]^ The scripts for tissue detection, color deconvolution, and intensity analysis are shown in Supplementary Table 2.

Autoradiography 

For the assessment of ^18^F-labeled CD73 tracer binding to human tissues, cryosections of breast tumor samples as well as sentinel lymph node samples were investigated. Cryosections were thawed and pre-incubated for 15 min at room temperature in 2-[4-(2-hydroxyethyl)piperazin-1-yl]ethanesulfonic acid buffer (HEPES). The ^18^F-labeled CD73 tracer was diluted to a radioactivity concentration of 1 MBq/mL. Blocking compounds were prepared according to the molar activity of the radiolabeled tracers [^18^F]MRS-4648 and [^18^F]PSB-19427 aiming at a 1000-fold excess of the blocking agent. For each tissue sample, neighboring sections were incubated for 20 min at room temperature with a mixture of 50µL of the radiotracer and 50µL of the classical CD73 inhibitor AMPCP (25 nmol, in PBS), or JMS0414 (61 nmol, in HEPES), or HEPES buffer respectively. After incubation, slides were washed three times with PBS and Aqua bidest, mounted on a slide holder for the image acquisition, and covered with protection and scintillation foils. Digital autoradiography measurement of each slide was performed for 60 minutes (Micro Imager, Biospace, France). Acquired data were corrected for radioactive decay in reference to the start of the acquisition as well as the start of the incubation.

Human tumor xenograft experiments 

MDA-MB-231 cells were cultivated in DMEM/GlutaMAX, supplemented with 10 % fetal calf serum, 100 U/mL penicillin, and 100 µg/mL streptomycin and L-glutamine to a final concentration of 8 mmol/L. AsPC-1 cells were cultivated in RPMI, supplemented with 10 % fetal calf serum, 100 U/mL penicillin, and 100 µg/mL streptomycin and L-glutamine to a final concentration of 8 mmol/L. 5 x 10^6^ MDA-MB-231 or 1x 10^6^  AsPC-1 cells were injected subcutaneously into the shoulder region of 7 to 12 weeks old NSG (NOD.Cg-*Prkdc*^*scid*^ *Il2rg*^*tm1Wjl*^/SzJ) mice (Charles River Laboratories). Two tumors were inoculated per animal, and growth was followed by digital caliper measurements (volume = ½ (length * width^2^)). 

In vivo imaging 

Adult C57bl/6 (biodistribution study, 19.8±1.3 g) or tumor-bearing NSG mice (22.2±2.2 g) were anesthetized by isoflurane/O_2_, and one lateral tail vein was cannulated using a 27 G needle. A respective radiotracer (~ 390 kBq/g body weight) was injected as a bolus *via* the tail vein, and subsequent PET scanning was performed. A ~1000-fold excess of unlabeled JMS0414 (**14** for [^18^F]MRS-4648 ([^18^F]**2**)), unlabeled PSB-19427 (**1**), or PSB-12651 (**15**) (for [^18^F]PSB-19427 ([^18^F]**1**)) was injected into a subgroup of tumor-bearing mice 10 min before radiotracer injection in the blocking studies. PET imaging studies were carried out using a submillimeter high resolution (0.7 mm full width at half-maximum) small animal scanner (32 module quadHIDAC, Oxford Positron Systems Ltd., Oxford, UK) with a uniform spatial resolution (<1 mm) over a large cylindrical field (165 mm diameter, 280 mm axial length). List-mode data were acquired for 90 min and reconstructed into dynamic time frames using an iterative reconstruction algorithm. In a subgroup of mice, measurements were complemented by late time point PET scans for 20 min 4 h after injection. Subsequently, after PET acquisitions, the scanning bed was transferred to the computed tomography (CT) scanner (Inveon, Siemens Medical Solutions, U.S.), and a CT acquisition with a spatial resolution of 80 μm was performed for each mouse. Final acquisitions were performed as contrast-enhanced CT with i.v. injection of iopromid (Ultravist®370). Reconstructed image data sets were coregistered based on extrinsic markers attached to the multimodal scanning bed, and the in-house developed image analysis software MEDgical (EIMI) was used for quantification. Three-dimensional volumes of interest (VOIs) were defined over the respective organs in CT data sets, transferred to the coregistered PET data, and analyzed quantitatively. Regional uptake was calculated as the percentage of injected dose by dividing counts per min (cpm) in the VOI by total counts in the mouse multiplied by 100 (%ID/mL). The clearance of the radiotracers was calculated based on the PET results. PET imaging studies using [^18^F]FDG were performed analogously with a few variations. List-mode data were acquired for 60-75 min after tracer injection and reconstructed using an iterative reconstruction algorithm. Additionally, the scanned mice remained alive after PET and CT scans and were used the next day for imaging experiments with [^18^F]PSB-19427 ([^18^F]**1**) for direct comparison of [^18^F]FDG  and [^18^F]PSB-19427 ([^18^F]**1**).

Ex vivo gamma counter measurements

Following the final PET-CT acquisition, 90 min p.i. or 260 min p.i., respectively, mice were euthanized by cervical dislocation, and a necropsy was performed. Ex vivo biodistribution of radioactivity was analyzed by scintillation counting (Wizard2 gamma counter, Perkin-Elmer Life Science) and the radioactivity in respective organs was decay-corrected and calculated as %ID per gram tissue (% ID/g).

Determination of kinetic rate index and estimation of residence time

Cell membrane preparations of MDA-MB-231 cells were performed as previously described.^[9]^ A dual point competition assay (at 20 min and 90 min) using [^3^H]PSB-17230 was employed for the determination of kinetic rate index (KRI) in analogy to a published procedure,^[60]^ determined at 25°C. The residence times were calculated by linear correlation based on a large database of previously determined KRI values and residence times of analogs. The following residence times were determined:

PSB-19427: 	22 min 
PSB-12651: 	31 min 
AMPCP: 	8.8 min

NMR data analysis

NMR spectra were processed with MestReNova 12.0 (MestreLab Research).

Statistical analysis

Statistical analyses of in vivo PET and ex vivo biodistribution data were performed using GraphPad Prism 7.0 or GraphPad Prism 10.2. Statistical analyses were performed using an unpaired t-test as indicated in the figure legends for each dataset.

NMR spectra, HPLC purity, HRMS spectra, FT-IR spectra of selected key intermediates

(2*R*,3*R*,4*S*,5*R*)-2-{2-Chloro-6-[(4-ethynylbenzyl)(propyl)amino]-9*H*-purin-9-yl}-5-(hydroxymethyl)tetrahydrofuran-3,4-diol (6)

^**1**^**H NMR:**

^**13**^**C NMR**:

[((2*R*,3*S*,4*R*,5*R*)-5-{2-Chloro-6-[(4-ethynylbenzyl)(propyl)amino]-9*H*-purin-9-yl}-3,4-dihydroxytetrahydrofuran-2-yl)methoxy] (phosphonomethyl)-phosphonic acid (7)

^**1**^**H NMR:**

^**13**^**C NMR:**

^**31**^**P NMR:**

 {[{[(2*R*,3*S*,4*R*,5*R*)-5-(2-Chloro-6-{4-[1-(2-fluoroethyl)-1*H*-1,2,3-triazol-4-yl) benzyl](propyl)amino}-9*H*-purin-9-yl)-3,4-dihydroxytetrahydrofuran-2-yl]methoxy{(hydroxy)phosphoryl]methyl}phosphonic acid (1, PSB-19427). 

^**1**^**H NMR:**

^**13**^**C NMR:**

^**19**^**F NMR:**

1-[(2*R*,3*R*,4*S*,5*R*)-3,4-Dihydroxy-5-(hydroxymethyl)tetrahydrofuran-2-yl]-4-[({4-[1-(2-fluoroethyl)-1*H*-1,2,3-triazol-4-yl]benzyl}oxy)imino]-3-methyl-3,4-dihydropyrimidin-2(1*H*)-one (11)

^**1**^**H NMR:**

^**13**^**C NMR:**

^**19**^**F NMR:**

**COSY:**

**gHSQC:**

**gHMBC:**

**HRMS:**

**FT-IR:**

**HPLC-Purity:**

{[{[(2R,3S,4R,5R) 5 ( 4 {[(4 Ethynylbenzyl)oxy]imino} 3 methyl 2 oxo 3,4-dihydropyrimidin 1(2H) yl) 3,4 dihydroxytetrahydrofuran 2-yl]methoxy}(hydroxy)phosphoryl]methyl}phosphonic acid (12)

^**1**^**H NMR:**

^**13**^**C NMR:**

**COSY:**

**gHSQC:**

**gHMBC:**

**HRMS:**

**FT-IR:**

**HPLC-Purity:**

[((2*R*,3*S*,4*R*,5*R*)-5-{4-[({4-[1-(2-Fluoroethyl)-1*H*-1,2,3-triazol-4-yl]benzyl}oxy)-imino]-3-methyl-2-oxo-3,4-dihydropyrimidin-1(2*H*)-yl}-3,4-dihydroxytetrahydro-furan-2-yl)methoxy]-(phosphonomethyl)phosphonic acid (MRS-4648 (2))

^**1**^**H NMR:**

^**13**^**C NMR:**

^**31**^**P NMR:**

^**19**^**F NMR:**

**COSY:**

**gHSQC:**

**gHMBC:**

**HRMS:**

**FT-IR:**

**Purity:**

References 

[9]	R. M. Idris, H. Al-Hroub, C. C. Schmies, P. Riziki, C. Renn, T. Claff, K. Sylvester, S. Moschütz, J. Reinhardt, W. Deuter-Conrad, J. M. Dietrich, M. Toma, B. K. Fleischmann, D. Wenzel, H. Zimmermann, M. Hölzel, N. Sträter, C. E. Müller, *Biomedicine & Pharmacotherapy* **2025**, *188*, 118115.

[23]	S. Bhattarai, J. Pippel, A. Meyer, M. Freundlieb, C. Schmies, A. Abdelrahman, A. Fiene, S.-Y. Lee, H. Zimmermann, A. El-Tayeb, G. G. Yegutkin, N. Sträter, C. E. Müller, *Advanced Therapeutics* **2019**, *2*, 1900075.

[28]	E. U. Sharif, J. Kalisiak, K. V. Lawson, D. H. Miles, E. Newcomb, E. A. Lindsey, B. R. Rosen, L. P. P. Debien, A. Chen, X. Zhao, S. W. Young, N. P. Walker, N. Sträter, E. R. Scaletti, L. Jin, G. Xu, M. R. Leleti, J. P. Powers, *J Med Chem* **2021**, *64*, 845-860.

[38]	M. Patberg, A. Isaak, F. Füsser, N. V. Ortiz Zacarías, L. Vinnenberg, J. Schulte, L. Michetti, L. Grey, C. van der Horst, P. Hundehege, O. Koch, L. H. Heitman, T. Budde, A. Junker, *Eur J Med Chem* **2021**, *226*, 113838.

[39]	K. Losenkova, M. Paul, H. Irjala, S. Jalkanen, G. G. Yegutkin, in *Purinergic Signaling: Methods and Protocols* (Ed.: P. Pelegrín), Springer New York, New York, NY, **2020**, pp. 107-116.

[41]	K. Losenkova, M. Zuccarini, M. Karikoski, J. Laurila, D. Boison, S. Jalkanen, G. G. Yegutkin, *J Cell Sci* **2020**, *133*.

[54]	M. Rafehi, J. C. Burbiel, I. Y. Attah, A. Abdelrahman, C. E. Müller, *Purinergic Signalling* **2017**, *13*, 89-103.

[55]	W. Kabsch, *Acta Crystallogr D Biol Crystallogr* **2010**, *66*, 125-132.

[56]	M. Krug, M. S. Weiss, U. Heinemann, U. Mueller, *J Appl Crystallogr* **2012**, *45*, 568-572.

[57]	P. Emsley, B. Lohkamp, W. G. Scott, K. Cowtan, *Acta Crystallogr D Biol Crystallogr* **2010**, *66*, 486-501.

[58]	C. J. Williams, J. J. Headd, N. W. Moriarty, M. G. Prisant, L. L. Videau, L. N. Deis, V. Verma, D. A. Keedy, B. J. Hintze, V. B. Chen, S. Jain, S. M. Lewis, W. B. Arendall Iii, J. Snoeyink, P. D. Adams, S. C. Lovell, J. S. Richardson, D. C. Richardson, *Protein Sci* **2018**, *27*, 293-315.

[59]	D. Liebschner, P. V. Afonine, N. W. Moriarty, B. K. Poon, O. V. Sobolev, T. C. Terwilliger, P. D. Adams, *Acta Crystallogr D Struct Biol* **2017**, *73*, 148-157.

[60]	P. D. Adams, P. V. Afonine, G. Bunkóczi, V. B. Chen, I. W. Davis, N. Echols, J. J. Headd, L. W. Hung, G. J. Kapral, R. W. Grosse-Kunstleve, A. J. McCoy, N. W. Moriarty, R. Oeffner, R. J. Read, D. C. Richardson, J. S. Richardson, T. C. Terwilliger, P. H. Zwart, *Acta Crystallogr D Biol Crystallogr* **2010**, *66*, 213-221.

[61]	D. Guo, E. J. H. van Dorp, T. Mulder-Krieger, J. P. D. van Veldhoven, J. Brussee, A. P. Ijzerman, L. H. Heitman, *SLAS Discovery* **2013**, *18*, 309-320.
